# Interferon-Inducible Mechanism of Dendritic Cell-Mediated HIV-1 Dissemination Is Dependent on Siglec-1/CD169

**DOI:** 10.1371/journal.ppat.1003291

**Published:** 2013-04-11

**Authors:** Wendy Blay Puryear, Hisashi Akiyama, Suzanne D. Geer, Nora P. Ramirez, Xinwei Yu, Björn M. Reinhard, Suryaram Gummuluru

**Affiliations:** 1 Department of Microbiology, Boston University School of Medicine, Boston, Massachusetts, United States of America; 2 Department of Chemistry and the Photonics Center, Boston University, Boston, Massachusetts, United States of America; Vanderbilt University School of Medicine, United States of America

## Abstract

Human immunodeficiency virus type 1 (HIV-1) interactions with myeloid dendritic cells (DCs) can result in virus dissemination to CD4^+^ T cells via a trans infection pathway dependent on virion incorporation of the host cell derived glycosphingolipid (GSL), GM3. The mechanism of DC-mediated trans infection is extremely efficacious and can result in infection of multiple CD4^+^ T cells as these cells make exploratory contacts on the DC surface. While it has long been appreciated that activation of DCs with ligands that induce type I IFN signaling pathway dramatically enhances DC-mediated T cell trans infection, the mechanism by which this occurs has remained unclear until now. Here, we demonstrate that the type I IFN-inducible Siglec-1, CD169, is the DC receptor that captures HIV in a GM3-dependent manner. Selective downregulation of CD169 expression, neutralizing CD169 function, or depletion of GSLs from virions, abrogated DC-mediated HIV-1 capture and trans infection, while exogenous expression of CD169 in receptor-naïve cells rescued GSL-dependent capture and trans infection. HIV-1 particles co-localized with CD169 on DC surface immediately following capture and subsequently within non-lysosomal compartments that redistributed to the DC – T cell infectious synapses upon initiation of T cell contact. Together, these findings describe a novel mechanism of pathogen parasitization of host encoded cellular recognition machinery (GM3 – CD169 interaction) for DC-dependent HIV dissemination.

## Author Summary

Dendritic cells (DCs) are one of the initial cellular targets of HIV-1 and can play a crucial role in determining the course of virus infection in vivo. While sentinel functions of DCs are essential for establishment of an antiviral state, HIV-1 can subvert DC function for its dissemination. One of the mechanisms by which DCs can mediate virus spread is via the trans infection pathway whereby DCs capture HIV-1 particles and retain them in an infectious state without getting infected, and pass these infectious particles to CD4^+^ T cells upon initiation of cellular contacts. In this report, we demonstrate that expression of Siglec-1or CD169, on DC surface is responsible for capture of HIV-1 particles by binding the ganglioside, GM3, present in the virion lipid bilayer. This interaction between CD169 and GM3 targets captured virus particles to non-degradative compartments and resulted in retention of virus particle infectivity within DCs. Upon initiation of T cell contacts with virus-laden DCs, HIV-1 particles were trafficked to the DC – T synaptic junctions and transferred to T cells for establishment of productive infection. These studies define a novel host-encoded receptor – ligand interaction that drives HIV-1 dissemination and can be used for development of novel anti-viral therapeutics.

## Introduction

Myeloid dendritic cells (DCs) are potent antigen presenting cells that monitor their immediate environment for invading pathogens. The ability of these sentinel DCs to sample and process antigen, and present processed antigen to naïve T cells is critical to the development of effective adaptive immune responses. HIV, in turn, has evolved to evade antigen presentation pathways and exploit DC biology to facilitate its dissemination within the infected host. DC-dependent HIV-1 trans infection of CD4^+^ T cells is an efficacious HIV dissemination mechanism [1], [2] that has been hypothesized to provide virus particles evasion from host innate and adaptive immune responses [3]. HIV-1 capture by DCs and access to the DC-dependent trans infection pathway has long been thought to be exclusively dependent on the interactions of the mannosylated virus envelope glycoprotein gp120 with C-type lectin receptors (CLRs) [4], such as dendritic cell-specific intercellular adhesion molecule-3-binding nonintegrin (DC-SIGN) [5]. In addition to HIV-1 capture, virus targeting to tetraspanin protein positive non-lysosomal compartments that allow for virus persistence and evasion from lysosomal degradation pathways in DCs [6], [7], [8] has also been reported to be dependent on DC-SIGN [9], [10].

In contrast, knock-down of DC-SIGN expression in DCs using shRNAs [11], or blocking DC-SIGN function using neutralizing antibodies [12], [13] have failed to attenuate DC-mediated HIV-1 capture or trans infection. Furthermore, while DCs upon maturation downregulate cell surface expression of DC-SIGN and CLRs, HIV-1 capture and trans infection efficiency is dramatically enhanced upon maturation over that observed with immature DCs [7], [14], [15]. Interestingly, capture of HIV-1 particles by DCs can occur in a gp120-independent manner, and this virus capture mechanism is also enhanced upon maturation of DCs [7], [14]. Furthermore, capture of gp120-deficient virus-like particles by mature DCs can result in localization within non-lysosomal compartments and trafficked to DC – T cell infectious synapses upon initiation of T cell contacts [7]. These findings highlight the existence of a virus particle associated host encoded HIV-1 capture mechanism in DCs.

In addition to gp120, HIV-1 particles also incorporate host cell derived constituents in their lipid bilayer. Assembly of HIV-1 particles within plasma membrane lipid rafts [16], [17] results in production of virions with a unique surface exposed host cell derived proteome and lipidome [18], [19], [20]. Interestingly, selective depletion of virion-associated glycosphingolipids (GSLs), either by targeting GSL biosynthesis pathways by small molecule inhibitors [7], [14] or by siRNAs targeting GSL biosynthetic enzymes in virus producer cells [21] can attenuate gp120-independent capture of HIV-1 particles by mature DCs. In support of this hypothesis, we and others, have recently demonstrated that gp120-independent capture of HIV-1 by DCs is dependent on the expression of the ganglioside, GM3 (α2,3-sialylated GSL) in the virus particle membrane [21], [22]. The data presented in this report demonstrate that the IFNα-inducible sialic-acid-binding immunoglobulin-like lectin (Siglec-1), CD169, is the receptor that mediates the gp120-independent, GM3-dependent interaction of HIV-1 with DCs and defines a novel molecular mechanism (virus particle associated GM3 binding by CD169) by which HIV-1 transits through the DC to arrive at the infectious synapse and may provide a novel conserved host-derived target (GM3 – CD169 interaction) for intervention strategies.

## Results

### DCs Express CD169 Whose Expression Is Enhanced upon Stimulation with IFN-α

**Figure 1:**
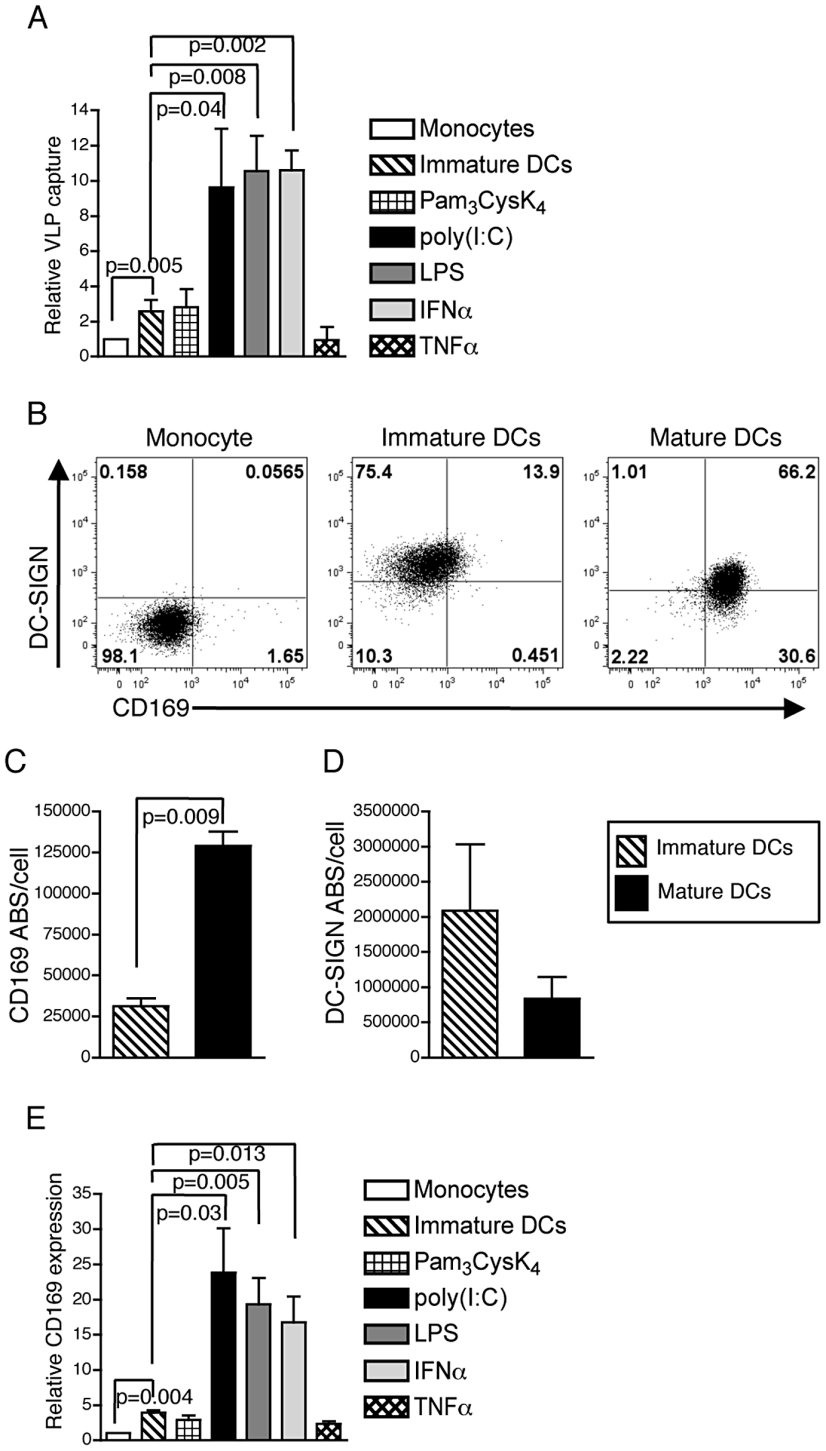
Dendritic cell activation by TRIF-dependent TLR ligands or IFNα induces CD169 expression and enhances HIV-1 capture. A. Capture of VLPs by monocytes, immature DCs or DCs matured with Pam_3_Cysk_4_, poly(I:C), LPS, IFNα, or TNFα was determined by FACS and is reported as relative capture by cells normalized to that observed with monocytes (mean ± SD). B. Representative expression of DC-SIGN and CD169 on monocytes, immature DCs (day 6 post differentiation), or DCs matured with LPS (last 2 days of differentiation). For each day, cells were gated based on staining with isotype. QFACS measurement of cell surface CD169 (C) and DC-SIGN (D) on immature and LPS-matured DCs. Shown are the average number of ABS per cell (mean ± SD). E. Relative cell surface expression of CD169 on immature DCs or DCs matured with Pam_3_Cysk_4_, poly(I:C), LPS, IFNα, or TNFα normalized to that observed with monocytes (set as 1), (mean MFI ± SD). The data is from cells derived from 3 independent donors and experiments performed in triplicate.

To identify the DC-receptor required for GM3-dependent HIV-1 capture, primary peripheral blood CD14^+^ monocytes, immature DCs, and DCs activated with TLR2 (Pam_3_CysK_4_), TLR3 (poly(I:C)), or TLR4 (E. coli LPS) agonists, or stimulated with the cytokines, IFNα or TNFα were tested for their ability to capture Env-deficient HIV-1 Gag-eGFP virus like particles (VLPs). While monocytes failed to capture VLPs, differentiation of monocytes into DCs resulted in a low level of VLP capture (Figure 1A). Furthermore, stimulation of immature DCs with TLR3 or TLR4 agonists dramatically enhanced VLP capture (Figure 1A), similar to the previously observed maturation induced enhancement in infectious virus capture by DCs [7], [14], [15]. But surprisingly, activation of DCs with Pam_3_CysK_4_ (TLR2 agonist) resulted in no appreciable increase in VLP capture over that observed with immature DCs (Figure 1A). While signaling downstream of all TLRs is conceptually similar and can result in maturation of DCs, individual TLRs trigger different signal transduction pathways with the key distinction being that TLR3 and TLR4 signaling can induce both pro-inflammatory and type I IFN responses via a TRIF-dependent pathway while TLR2 receptors induce only a pro-inflammatory response [23]. Interestingly, direct exposure of immature DCs to IFNα alone but not TNFα, resulted in a similar level of VLP capture to that observed with LPS or poly(I:C)-stimulated DCs (Figure 1A), suggesting HIV-1 capture by DCs is dependent on the presence of an IFNα-inducible factor.

We and others have demonstrated that capture of HIV-1 by LPS-matured DCs is dependent on virion incorporation of terminal α2,3-sialylated GSL, GM3 [21], [22]. Interestingly, this IFN-inducible, GSL-dependent capture mechanism of VLPs was specific to myeloid cells and was exquisitely sensitive to proteolytic cleavage of DC surface expressed proteome (Supplementary Figure S1). The sialoadhesin, Siglec1 or CD169, a member of the sialic acid binding immunoglobulin superfamily of lectins [24], is a type I IFN-inducible myeloid cell specific protein, with binding specificity for gangliosides that contain terminal α2-3 linked terminal sialic acid residues, such as GM3 [25]. While peripheral blood CD14^+^ monocytes do not express CD169 (Figure 1B), expression on monocytes can be induced upon exposure to IFNα in vitro and in vivo [26], [27], [28]. Interestingly, some studies have also reported inducible expression of CD169 on DCs in vitro [29]. We sought to determine if peripheral blood monocyte-derived DCs express CD169 and whether CD169 functions as the DC receptor responsible for GSL-dependent capture of HIV-1. Differentiation of monocytes to immature DCs resulted in a low level of CD169 expression by day 6 post-initiation of differentiation (∼25,000 ABS/cell; Figure 1B and C), with a dramatic enhancement in cell surface CD169 expression upon subsequent stimulation with LPS (∼125,000 ABS/cell; Figure 1B and C). In contrast cell surface expression of DC-SIGN was inversely correlated with VLP capture phenotype, in that immature DCs expressed high levels of DC-SIGN (>2,000,000 ABS/cell; Figure 1B and D), while cell surface DC-SIGN expression was downregulated upon maturation with LPS (∼600,000ABS/cell; Figure 1B and D). It should be noted that measurement of cell surface DC-SIGN expression by QFACS is an approximation, since DC-SIGN ABS values fell beyond the linear range of the standards employed in the assay, and, hence were derived via extrapolation of the standard curve beyond the highest standard (300,000 ABS). Interestingly, in strict correlation with the observed type I IFN-dependent enhancement of VLP capture by DCs (Figure 1A), enhancement of CD169 expression was only observed upon stimulation of immature DCs with poly(I:C), LPS or IFNα but not with Pam_3_CysK_4_ or TNFα (Figure 1E),

### Blocking CD169 on DCs Attenuates HIV-1 Capture and Trans Infection

**Figure 2:**
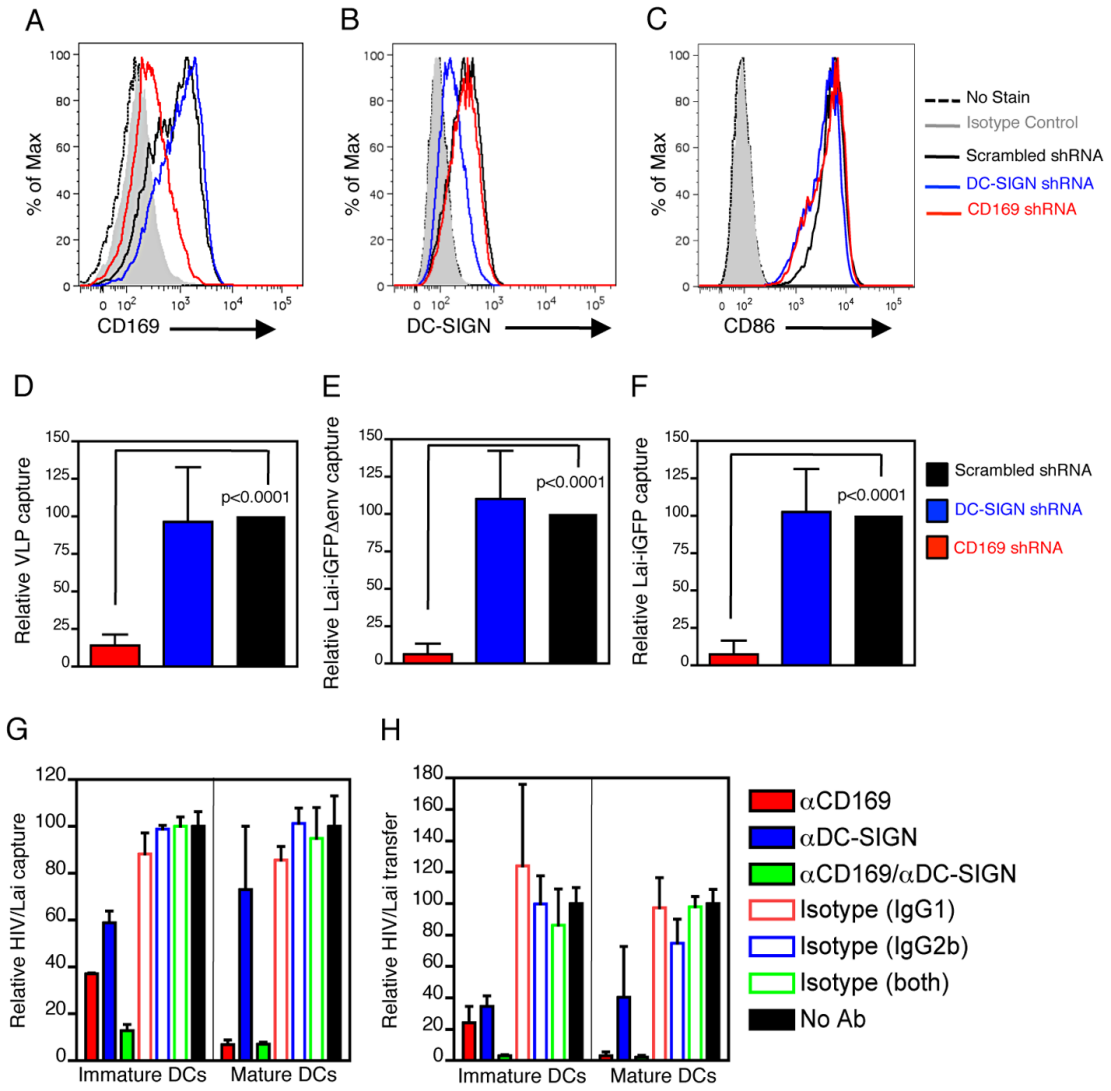
CD169 mediates HIV-1 capture and trans infection by DCs. Representative FACS analysis of CD169 (A), DC-SIGN (B), or CD86 (C) expression on DCs transduced with lentivectors expressing scrambled sequence, CD169 or DC-SIGN shRNAs. Relative capture by DC-SIGN or CD169 shRNA expressing DCs to that observed with scrambled shRNA expressing DCs of HIV Gag-eGFP VLPs (D), HIV/Lai-iGFPΔenv (E), and HIV/Lai-iGFP (F) is reported. DC mediated HIV/Lai capture (G) and transfer to autologous CD4^+^ T cells (H) ± isotypes or nAbs against CD169, DC-SIGN, or both. Shown is the average ± SD of cells derived from at least 4 donors (A through F) or one of two experiments done in triplicate (G and H).

To determine if capture of HIV-1 by mature DCs is dependent on CD169 specifically, immature DCs were transduced with lentivectors expressing CD169-specific shRNA, DC-SIGN-specific shRNA or scrambled control shRNA prior to initiation of maturation with LPS. CD169 expression was effectively suppressed upon transduction with CD169-shRNA (∼80% decrease in cell surface CD169 expression; (Figure 2A and Supplementary Figure S2A, C), while DC-SIGN cell surface expression was downregulated (∼50%) upon transduction with DC-SIGN shRNA (Figure 2B, and Supplementary Figure S2B, D), though transduction with either shRNA had negligible impact on cell surface expression of CD86 (Figure 2C). Interestingly, knock-down of CD169 expression on DCs robustly inhibited capture of VLPs (Figure 2D), Env-deficient HIV-1 (Figure 2E), or infectious HIV-1 particles (Figure 2F), while decrease in DC-SIGN had negligible impact on virus capture by mature DCs (Figure 2D, E and F).

To achieve high efficiency transduction of immature DCs with shRNA expressing lentivectors, pretreatment with SIV_mac_ Vpx containing VLPs was required that resulted in low level of DC maturation (data not shown). Hence, we utilized neutralizing antibodies (nAb) to define the role of CD169 in HIV-1 capture by immature and mature DCs. Pre-exposure to α-CD169 nAb alone blocked virus capture (Figure 2G) and trans infection to autologous CD4+ T cells (Figure 2H) by immature and mature DCs, while α-DC-SIGN nAb alone had modest inhibitory effects in immature DCs and failed to block HIV-1 capture by mature DCs (Figure 2G and H), consistent with previously published results [11], [12], [13], [30]. While pre-incubation with a combination of α-DC-SIGN and α-CD169 nAbs completely abrogated HIV-1 capture and trans infection by immature DCs, there was no additional attenuation observed with the combination of α-DC-SIGN and α-CD169 antibodies to that observed with α-CD169 antibody alone in mature DCs (Fig. 2G and H). Though DCs also express additional Siglecs, such as Siglec-7 and -9 with specificity for α2-8 and α2-3 sialic acid containing glycans, respectively [24], capture of VLPs by mature DCs was only inhibited upon pre-incubation with CD169 nAb but not α-Siglec-7 or α-Siglec-9 nAbs, even upon desialylation of DC surface (Supplementary Figure S3), that can result in unmasking of sialic acid binding sites on Siglecs [24]. These results suggest that CD169 is largely responsible for DC-mediated HIV capture and trans infection.

### Co-localization of CD169 and HIV-1 Particles within DCs and at the DC – T Cell Infectious Synapse

**Figure 3:**
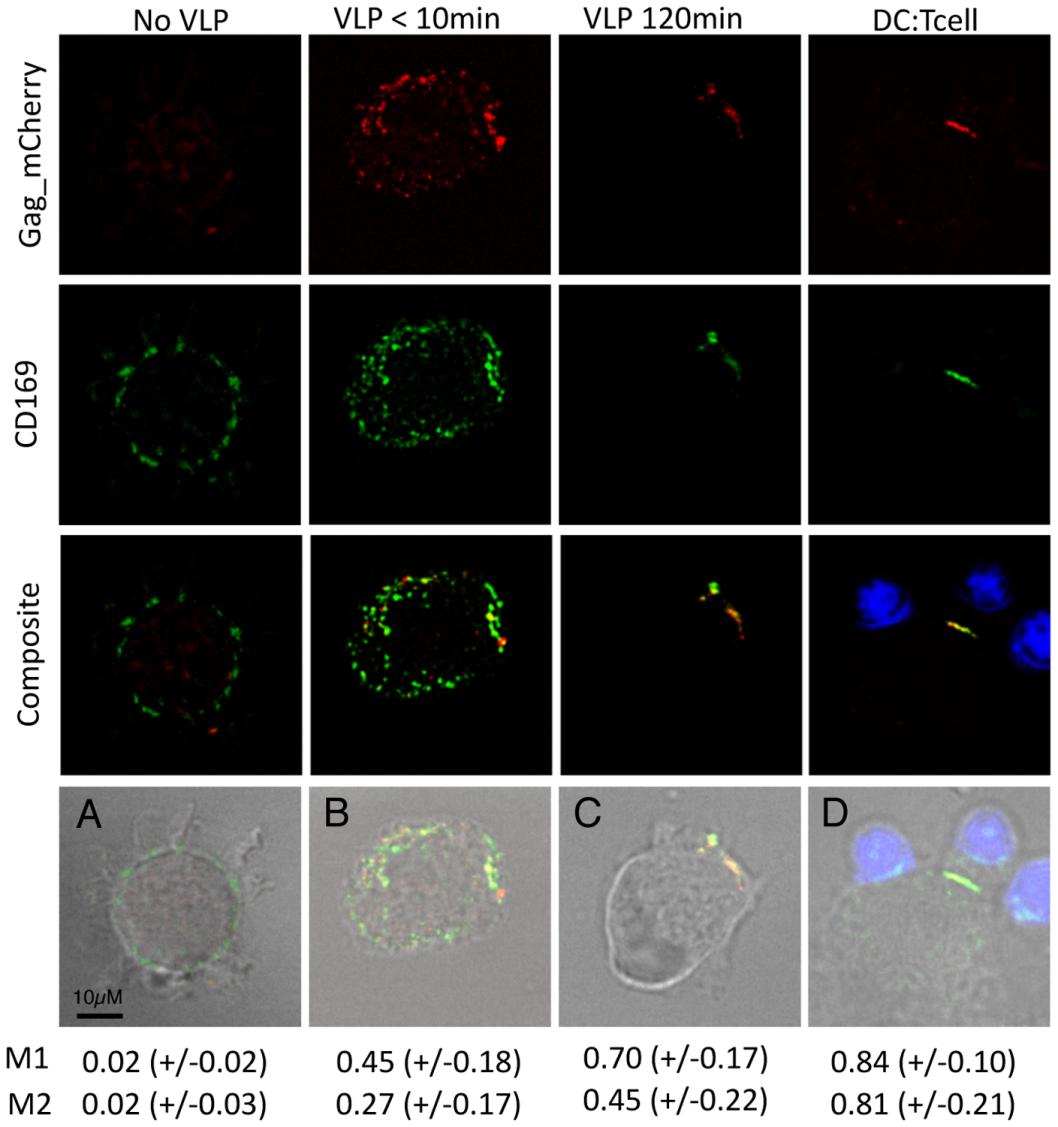
Captured HIV-1 particles co-localize with CD169 on DCs. Mature DCs were either (A) mock infected or exposed to Gag-mCherry VLP for (B) <10 minutes, (C) 120 minutes, or (D) 60 minutes with an additional 60 minute co-culture with autologous CD4^+^ T cells. Representative deconvolved maximum intensity images are shown. Top row = Gag-mCherry (red), second row = CD169 (green), third row = composite images (T cells in blue), bottom row = bright-field. Co-localization is reported as the average Mander's coefficient ± SD. M1 = ratio of Gag-mCherry associated with CD169, M2 = ratio of CD169 that has associated Gag-mCherry.

Capture of HIV-1 by mature DCs can result in sequestration of virus particles in teraspanin positive non-lysosomal compartments, that might be connected to the DC surface by deep membrane invaginations, and transferred to T cells upon initiation of DC – T cell contacts [2], [6], [7], [8], [31]. To determine if CD169 mediates HIV-1 trafficking to DC – T cell infectious synapses, cell surface distribution of CD169 on mature DCs was determined by confocal microscopy. While CD169 distribution on mature DCs displayed punctate staining in the absence of virus (Figure 3A), there was clear evidence of redistribution of the receptor within 10 min of exposure to HIV Gag-mCherry VLPs, with 27% of CD169 associated with virus particles (Figure 3B). By 120 min post exposure, CD169 underwent a dramatic polarization (Figure 3C) that resulted in 81% of CD169 co-localized with VLPs at the DC – T cell infectious synapse (Figure 3D). HIV Gag-mCherry VLPs showed nearly 45% co-localization with CD169 by 10 min post exposure (Figure 3B), and increasing after 2 h of virus exposure to 70% or 84% in the absence (Figure 3C) or presence (Figure 3D) of autologous CD4^+^ T cells, respectively. We verified similar patterns of co-localization between CD169 and infectious virus particles (Lai-iGFP) and again observed the spatial concentration coincident between CD169 and viral particles within 2 h post virus exposure (Supplementary Figure S4A and B).

While CD169 has been hypothesized to phagocytose sialylated pathogens [32], mechanism of endocytosis remains unclear. Interestingly, HIV-1 particles were trafficked to non-lysosomal compartments at the cell periphery, since no co-localization was observed between VLPs and endosomal markers, EEA1 and LAMP1 (Supplementary Figure S4D and E), nor between VLPs and cell surface proteins CD9 and CD45, at 10 min or 120 min post virus exposure (Supplementary Figure S4C, F and G). These results suggest that interaction of HIV-1 particles with CD169 on mature DC surface leads to virus particle localization at the cellular periphery that might correspond to the previously described deep plasma membrane invaginations [8], [31] and upon initiation of DC – T cell contacts, trafficked to the DC – T cell infectious synapse.

### CD169 Is the HIV-1 Trans Infection Factor on Inflammatory and Myeloid DCs

**Figure 4:**
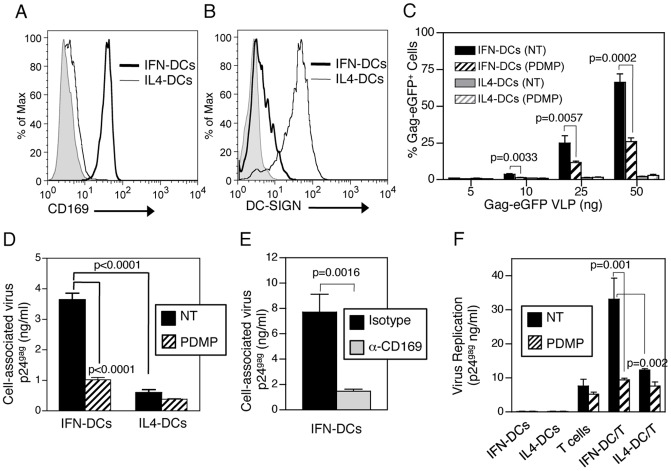
CD169 is the GSL-dependent HIV-1 attachment factor on inflammatory DCs necessary for virus capture and trans infection. Representative FACS analysis of CD169 (A) and DC-SIGN (B) expression on IFN-DCs or IL-4 DCs. The shaded histograms represent isotype controls. Capture of untreated (NT) or GSL-depleted (PDMP) HIV Gag-eGFP VLPs by DCs was determined by FACS (C). Capture of untreated (NT) or GSL-depleted (PDMP) HIV/Lai-Bal (D) or virus capture in the presence of isotype or α-CD169 (10 µg/ml) nAb (E) was determined by measuring cell-associated p24^gag^. HIV/Lai-Bal (±PDMP) replication in DCs, CD4^+^ T cells, or DC – T cell co-cultures was determined by measuring p24^gag^ in supernatants harvested 2 days post infection (F). Average ± SD of one of 3 independent experiments done in triplicate.

Even though IL-4/GM-CSF pathway of DC differentiation from peripheral blood monocytes is an efficient in vitro method to generate immature DCs [33], IL-4 is an anti-inflammatory cytokine [34] and high levels of IL-4 are unlikely to be present during host response to acute virus infections. In contrast, during acute SIV infection, a rapid influx of type I IFN-producing plasmacytoid DCs into the genital mucosa has been noted [35]. Furthermore, under conditions of chronic immune activation as a consequence of a compromised gut epithelial barrier, a hallmark of HIV-1 infection in vivo, a low-level of circulating type I IFNs has been observed [36]. Such inflammatory conditions provide strong signals for rapid influx and differentiation of monocytes into tissue inflammatory DCs [37], [38], [39], [40]. Interestingly, peripheral blood CD14^+^ monocytes stimulated with IFNα and GM-CSF are differentiated into inflammatory DCs (referred to as IFN-DCs) that can successfully prime naïve T cells and bias the T helper response towards a Th1 type [41], [42]. We sought to determine the interactions of HIV-1 particles with DCs differentiated from monocytes under pro-inflammatory conditions. Interestingly, IFN-DCs expressed high levels of CD169 (150,060±49,827 ABS/cell) but not DC-SIGN (20,298±16,147 ABS/cell) (Figure 4A and Supplementary Figure S5A), in contrast to DCs differentiated in the presence of IL-4 and GM-CSF for 3 days (IL4-DCs) that expressed high levels of DC-SIGN (1,848,000±364,359 ABS/cell) with little to no expression of CD169 (20,040±3,604 ABS/cell) (Figure 4B and Supplementary Figure S5B). While there was some variation in the expression of CD169 and DC-SIGN between donors, these two DC populations provided phenotypically distinct subtypes that expressed either CD169 or DC-SIGN.

CD169^+^ IFN-DCs, but not DC-SIGN^+^ IL4-DCs captured VLPs in a dose-dependent manner (Figure 4C). Furthermore, depletion of GSLs from virus particles via PDMP (competitive inhibitor of glucosylceramide synthase) treatment of virus producer cells (Supplementary Figure S6) [14], resulted in attenuation of capture of both VLPs and HIV/Lai-Bal virus particles by IFN-DCs (Figure 4C and D). Interestingly, capture of VLPs and HIV/Lai-Bal particles by DC-SIGN^+^ IL4-DCs was significantly lower in comparison to that exhibited by CD169^+^ IFN-DCs (Figure 4C and D). Cellular source of virus has been hypothesized to play a significant role in the ability of HIV-1 particles to interact with DC-specific attachment factors [14], [43], [44]. The virus producer cell phenotype had no impact, since capture of HIV/Lai-Bal derived from either HEK293T cells or PBMCs by IFN-DCs was dependent on GSLs (Figure 4D and Supplementary Figure S7), in a CD169-dependent manner (Figure 4E). Even though IFN-DCs expressed CD4 and CCR5 (Supplementary Figure S5A), these cells were refractory to productive infection (Figure 4F). Rather, captured particles were transferred to autologous CD4^+^ T cells, with a greater efficiency than IL4-DCs (Figure 4F), and with a stringent requirement for GSLs in the virus particle membrane (Figure 4F).

**Figure 5:**
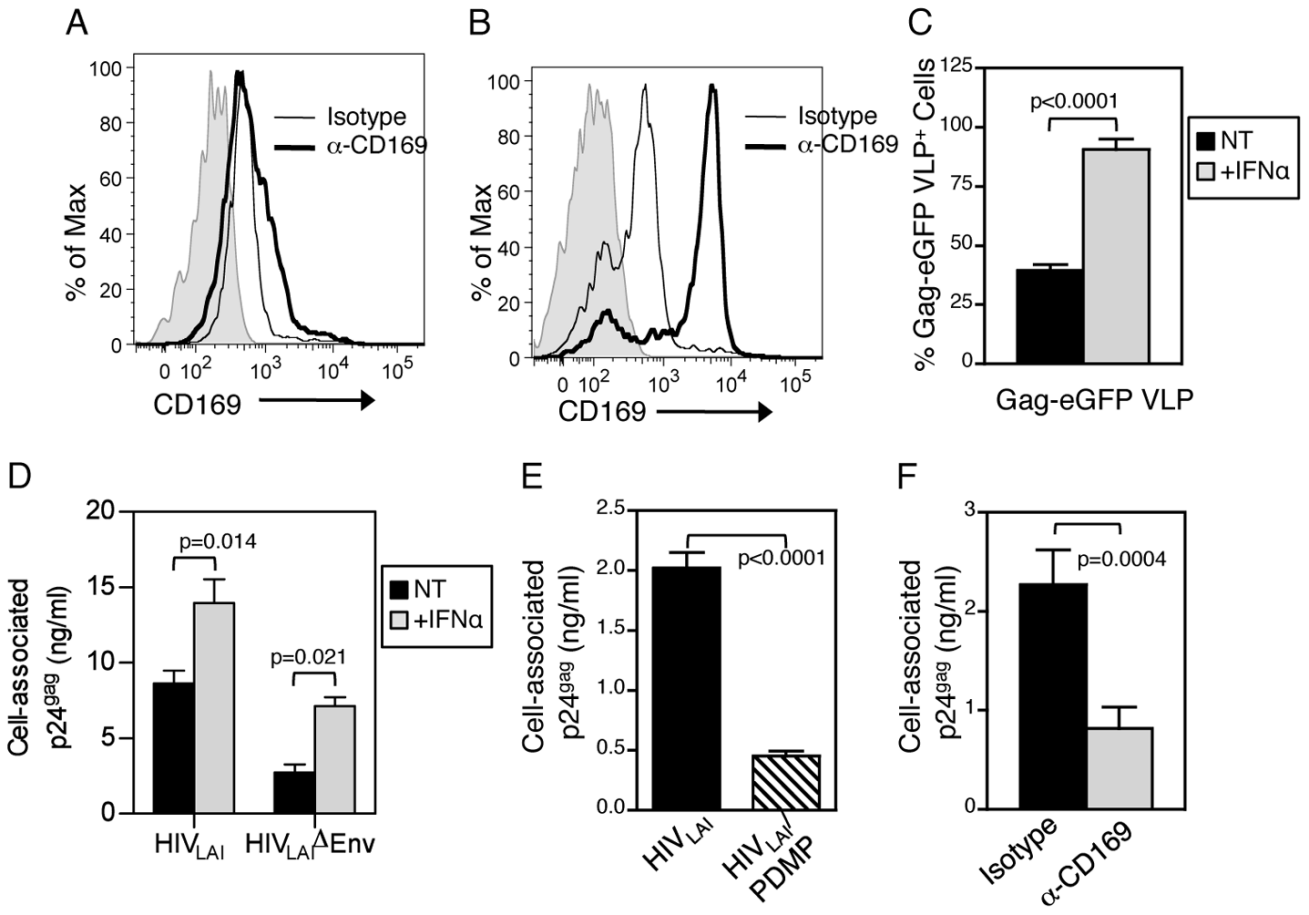
Peripheral blood myeloid DCs require CD169 for HIV-1 capture. CD169 expression was determined for (A) untreated and (B) IFNα matured myeloid DCs by FACS. Shaded histograms represent unstained cells. Capture of HIV Gag-eGFP VLPs (C) and HIV/Lai or HIV/LaiΔenv (D) by untreated (NT) or IFNα-exposed myeloid DCs was determined by FACS and ELISA, respectively. Capture of GSL-depleted (PDMP) or untreated HIV/Lai particles (E) in the presence of isotype or α-CD169 nAb (F) by IFNα exposed myeloid DCs was determined by measuring cell-associated p24^gag^. Average ± SD of one of three independent experiments done in triplicate.

In addition to monocyte-derived DCs, we sought to determine if CD169 dependent interactions of DCs with HIV are also conserved in circulating blood DCs. since previous studies have demonstrated DC-SIGN or CLR-independent capture and trans infection of HIV-1 by these cells [45], [46]. BDCA1^+^ myeloid DCs directly isolated from PBMCs expressed CD169 (Figure 5A), and expression of CD169 was enhanced upon stimulation with IFNα (Figure 5B). Capture of VLPs (Figure 5C), Env-deficient, or infectious HIV-1 particles (Figure 5D) by myeloid DCs was also increased upon IFNα stimulation, while challenge with GSL-depleted particles (Figure 5E) or pre-incubation with α-CD169 nAb (Figure 5F) attenuated capture. These results suggest that HIV capture and access to the trans infection pathway by both inflammatory and myeloid DCs is dependent on the expression of CD169 on the cell surface and GSLs in the virus particle membrane.

### Constitutive Expression of CD169 in Receptor-Naïve Cells Rescues Env-independent-HIV-1 Capture and Trans Infection

**Figure 6:**
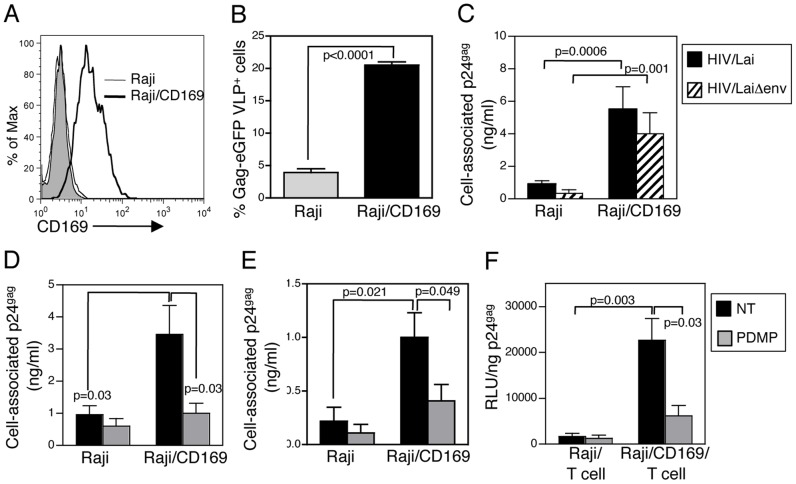
Exogenous expression of CD169 rescues GSL-dependent HIV-1 capture and trans infection. A. CD169 expression on Raji and Raji/CD169 cells was determined by FACS. Shaded histogram represents staining with isotype control. Capture of HIV Gag-eGFP VLP (B) or HIV/Lai and HIV/LaiΔenv (C) by Raji and Raji/CD169 cells was determined by FACS and p24^gag^ ELISA, respectively. Capture of HEK293T-derived (D) or PBMC-derived (E) HIV/Lai-Bal (±PDMP) by Raji and Raji/CD169 cells was determined by measuring cell-associated p24^gag^. F. Raji or Raji/CD169-mediated trans infection of HIV/Lai-Bal-luc (±PDMP) was determined by measuring luciferase activity in Raji/T cell co-cultures 2 days post-infection. Data reported is from one experiment performed in triplicate (mean ± SD) and is representative of 5 independent experiments.

To determine if CD169 expression alone is sufficient for mediating HIV-1 capture and trans infection, CD169-deficient Raji B cells were engineered to constitutively express CD169 (Figure 6A). While parental Raji cells did not bind Env-deficient HIV-1 particles (Figure 4B) [5], [47], CD169 expression rescued the ability of these cells to capture VLPs (Figure 6B), and Env-deficient or infectious HIV-1 particles (Figure 6C). Furthermore, exogenous expression of CD169 on Raji cells resulted in efficient capture of HEK293T (Figure 6D) and PBMC-derived virus particles (Figure 6E), and capture efficiency was significantly attenuated upon GSL depletion from virus particle membrane (Figure 6D and E). Interestingly, Raji/CD169 cells efficiently transferred captured HIV/Lai-Bal virus particles to CD4^+^ T cells in a GSL dependent manner (Figure 6F). These results suggest that expression of CD169 alone defines the ability of DCs to capture and transfer HIV-1 particles.

### α2,3-Sialic Acid Recognition Is Essential for CD169-Mediated HIV Capture and Trans Infection

**Figure 7:**
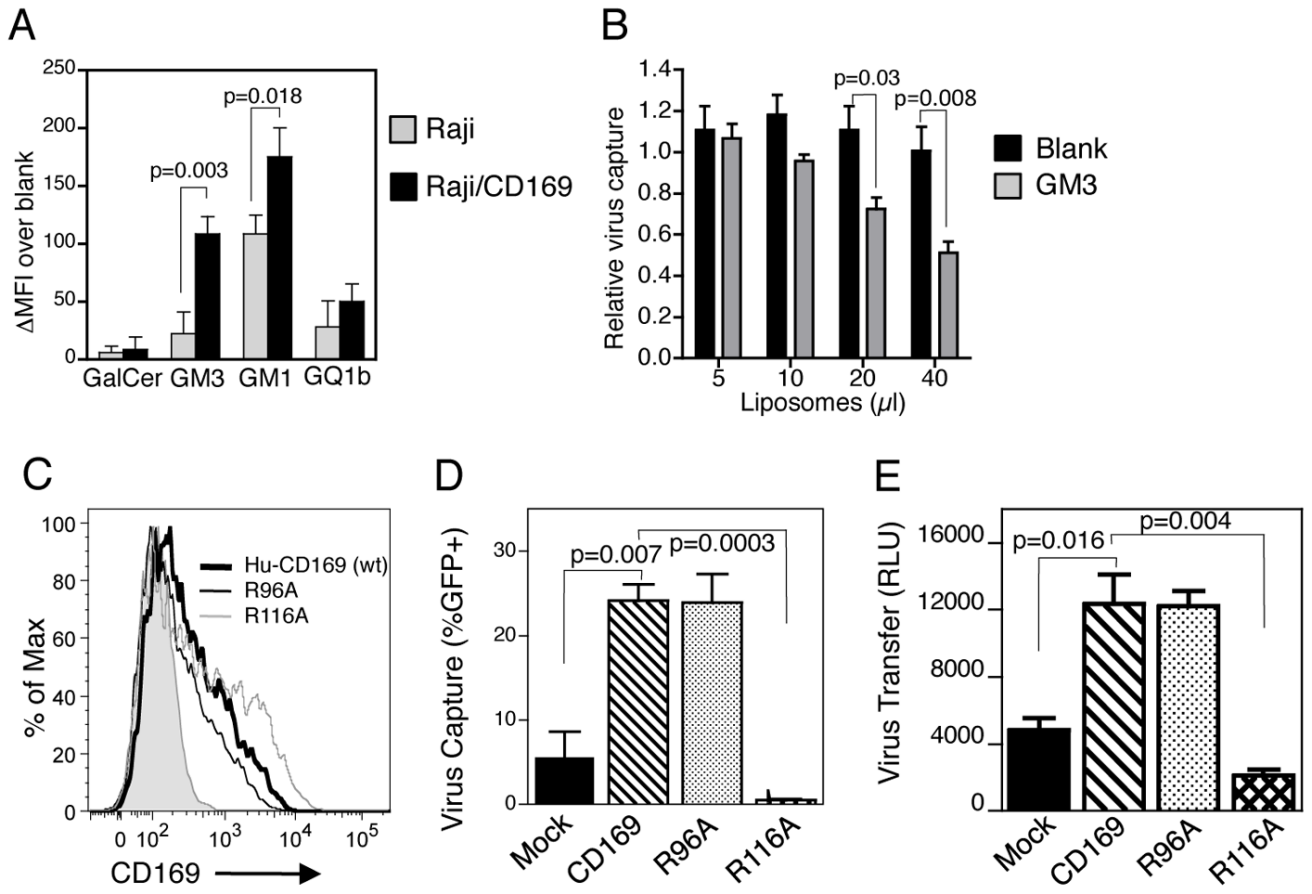
Recognition of α-2,3 sialylated GSL, GM3, by CD169 is essential for HIV-1 capture and trans infection. A. Capture of fluorescent liposomes by Raji and Raji/CD169 cells was determined by FACS, and is reported as the change in MFI upon challenge with phospholipid-liposome over blank liposomes and is the average MFI ± SD. B. Competitive inhibition of virus capture by Raji/CD169 cells by blank or 1% GM3 containing liposomes was determined by measuring cell-associated p24^gag^. C. Expression of human CD169 and mutants in HEK293T cells determined by FACS. Shaded histogram represents staining with isotype control. Capture of HIV/Lai-iGFP (D) and transfer of Lai-Balenv/luc+ (E) by HEK293T cells expressing wildtype-CD169 or mutants (R96A or R116A) were determined by FACS analysis (for %GFP+ cells) and luciferase activity in co-cultures (HEK293T/CD4+ T cells), respectively. The data shown is from one experiment, performed in triplicate (mean ± SD), and is representative of 3 independent experiments.

Our previous studies had demonstrated binding of GM3 and GM1 (terminal α2,3 sialic acid residue containing GSLs) liposomes by mature DCs [21]. In additional support of a role for CD169 as the primary attachment factor on DCs responsible for α2,3 sialylated GSL recognition, Raji/CD169 cells preferentially bound GM3 or GM1 containing liposomes, over that observed with parental Raji cells (Figure 7A), while no enhancement in liposome binding was observed with Raji/CD169 cells compared to Raji cells upon challenge with liposomes containing either GalCer or GQ1b (terminal α2,8 sialic acid residues) (Figure 7A). Furthermore, Env-deficient HIV-1 capture by Raji/CD169 cells was competitively inhibited by GM3-containing liposomes (Figure 7B), similar to previous findings with mature DCs [21].

The extracellular region of CD169, like other members of the Siglec family, consists of a single V-set immunoglobulin (Ig)-like domain that contains the sialic acid binding motif [24], [48]. We mutated the critical Arg residue in the extracellular N-terminal V-set domain (R116A) shown previously to be essential for sialic acid binding by Siglecs [25], [48], and a control Arg residue (R96A), not predicted to be required for sialic acid recognition. Induced expression of CD169 in HEK293T cells (Figure 7C and Supplementary Figure S8A) rescued VLP (Supplementary Figure S8B) and infectious virus capture (Figure 7D). Though total and cell surface expression of both mutants (R96A and R116A) was equivalent to that of wild type CD169 in transiently transfected HEK293T cells (Supplementary Figure S8A and Figure 7C), there was a complete loss of VLP (Supplementary Figure S8B) and infectious HIV-1 capture (Figure 7D) and transfer (Figure 7E) by only the sialic acid binding deficient CD169/R116A mutant.

### Conservation of GSL-dependent Retroviral Capture Function by Human and Murine CD169

**Figure 8:**
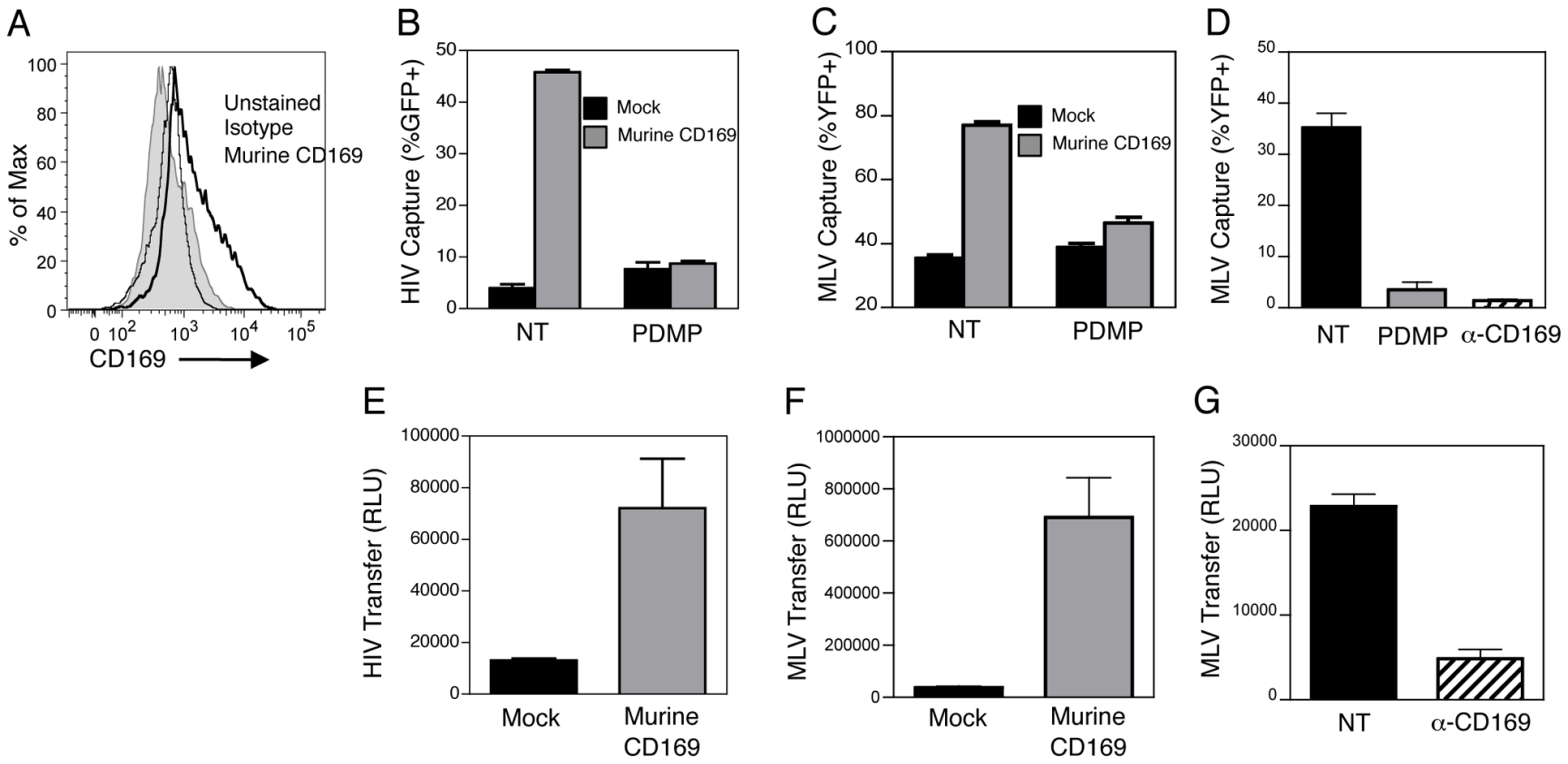
Retroviral capture and transfer function is conserved between human and mouse CD169. A. Expression of murine CD169 on HEK293T cells determined by FACS. Capture of untreated (NT) or GSL-depleted HIV Gag-eGFP VLPs (B) or MLV Gag-YFP VLPs (C) by mock transfected or murine CD169 expressing HEK293T cells was determined by FACS. D. Capture of untreated (NT), or GSL-depleted (PDMP) MLV Gag-YFP VLPs, or capture of MLV Gag-YFP VLPs, in the presence of α-CD169 nAb by mDCs was determined by FACS. E. HEK293T/murine CD169 mediated trans infection of Lai-Balenv/luc^+^ to CD4+ T cells (E), or transfer of MLV-E/luc to Rat-2 cells (F) was determined by measuring luciferase activity in HEK293T/CD4^+^ T cell and HEK293T/Rat-2 co-cultures, respectively, 2 days post-infection. G. Transfer of MLV-E/luc by mDCs in the presence or absence of α-CD169 nAb was determined by measuring luciferase activity in mDC/Rat-2 cell co-cultures 2 days post-infection. The data shown is from one experiment, performed in triplicate (mean ± SD), and is representative of 2 independent experiments.

Siglec1 or CD169 is extremely well-conserved amongst various mammalian species, with the highest similarity observed in the N-terminal extracellular domain [24]. In particular, amino acids in the V-set Ig-like domain essential for sialic acid recognition are identical between human and mouse CD169 proteins [25]. Interestingly, similar to HIV-1 particles, GM3 is also enriched in the lipid bilayer of the simple retrovirus, murine leukemia virus (MLV) [18]. Furthermore, capture of HIV-1 particles by mature DCs can be competitively inhibited by ecotropic-MLV particles [7]. Since animal models can prove invaluable for the study of CD169 function in vivo, we wanted to determine if murine CD169 could also function as a retroviral attachment factor that can mediate GM3-dependent virus capture and transfer. Transient expression of murine CD169 in HEK293T cells resulted in high cell surface expression (Figure 8A) and robust HIV Gag-eGFP (Figure 8B) and MLV Gag-YFP VLP capture (Figure 8C). To determine if capture of MLV Gag-YFP VLPs was also dependent on GSLs, GSL-depleted MLV Gag-YFP VLPs were produced from PDMP-treated HEK293T cells (Supplementary Figure S9A and B). Interestingly, capture of retroviral VLPs by murine CD169 was also dependent on presence of GSLs in the virus particle membranes (Figure 8B and C). Furthermore, mature DCs (Figure 8D) and Raji/CD169 cells (Supplementary Figure S9D) could also capture MLV Gag-YFP VLPs in a GSL and human CD169-dependent manner.

We next determined if both human and murine CD169 could transfer captured HIV-1 particles to CD4^+^ T cells and MLV particles to murine cells, a mechanism of retroviral trans infection. HEK293T cells transiently transfected with human or murine CD169 were exposed to single cycle of replication competent luciferase expressing HIV-1/Lai-Bal or ecotropic-MLV (MLV-E) particles, washed and then co-cultured with human CD4^+^ T cells or MLV-E susceptible Rat-2 cells. Interestingly, we observed robust transfer of HIV-1 particles to CD4^+^ T cells (Figure 8E) and transfer of ecotropic MLV particles to Rat-2 cells (Figure 8F) by murine CD169. Finally, mDCs also transferred MLV-E particles to Rat-2 cells and this transfer mechanism was dramatically decreased if capture of MLV-E particles by mDCs was carried out in the presence of anti-CD169 blocking antibody prior to initiation of co-cultures (Figure 8G). Collectively, these results suggest that recognition of α2,3 sialylated GSL, GM3, on retroviral particles by CD169 is essential for access to DC-mediated retrovirus trans infection pathway.

## Discussion

The results presented in this report define a novel mechanism of type I IFN-dependent enhancement of HIV-1 capture by DCs and subsequent access to the DC-mediated trans infection pathway. Maturation of DCs with TLR ligands that induce TRIF-dependent type I IFN-signaling pathway, differentiation of monocytes to DCs in the presence of IFNα, or exposure of blood myeloid DCs to IFNα, resulted in enhanced expression of a HIV-1 attachment factor, CD169, that facilitated explosive virus replication in DC – CD4^+^ T cell co-cultures. In agreement with our findings, Izquierdo-Useros, et al, also reported (while our manuscript was under review) that Env-independent, ganglioside-dependent capture of HIV-1 particles by LPS-matured DCs required CD169 expression [49].

These findings are in direct contrast to a long-standing hypothesis in the literature that has suggested an absolute requirement for DC-SIGN in mediating DC-mediated HIV-1 trans infection [5]. While exogenous expression of DC-SIGN in B cell lines does result in robust HIV-1 trans infection [5], [47], whether DC-SIGN is the HIV trans infection factor in DCs has been a matter of considerable debate [11], [12], [13], [14], [45]. Immature DCs express high levels of DC-SIGN (Figure 1C), but most of the particles captured by immature DCs have previously been reported to be endocytosed rapidly and degraded [50], [51], and targeted towards antigen presentation pathways in a DC-SIGN dependent manner [52], [53]. The results in this study also failed to confirm a significant role for DC-SIGN in immature DC-mediated HIV-1 trans infection (Figure 2G), in agreement with previously published studies that have observed at best a two-fold attenuation in immature DC mediated virus capture and trans infection upon virus exposure in the presence of anti-DC-SIGN neutralizing antibodies [12], [13], [54], [55], [56]. In contrast, knock-down of DC-SIGN expression nor blocking DC-SIGN function with neutralizing antibodies in mature DCs had no impact on virus capture (Figure 2). Furthermore, maturation of DCs with LPS resulted in downregulation of DC-SIGN (Figure 1), but an upregulation of CD169 and a significant enhancement in maturation-dependent HIV-1 capture and trans infection. It is possible that localization of DC-SIGN itself at the immature DC – T cell immunological synapse [54] for generation of stable synaptic junctions is necessary for optimal immature DC, but not mature DC-mediated HIV-1 trans infection. While CD169 is essential for HIV-1 capture by mature DCs, whether CD169 is also necessary for the formation of stable mature DC – T cell synaptic junctions remains to be determined.

In contrast to trans infection, DC-SIGN has also been thought to enhance productive infection of DCs [51], [57], [58], [59]. Indeed, DC-SIGN dependent recognition of HIV gp120 might concentrate HIV-1 particles on the DC surface such that the probability of virus interaction with CD4 and CCR5 and productive infection in cis is enhanced [57], [60], though the potential benefit to HIV-1 replication is questionable. In addition to the observation that HIV-1 fusion to DCs dramatically declines upon maturation [61], intracellular environment of DCs is antagonistic to productive virus replication, presumably because of presence of SAMHD1 and other yet-to-be defined IFN-induced myeloid cell-specific anti-viral factors [62], [63], [64], [65]. In contrast, HIV-1 interactions with CD169 target captured virus particles to the trans infection pathway that is amenable to robust virus replication. Thus, incorporation of sialylated GSLs in the virus particle membrane, we hypothesize, is a unique example of molecular mimicry by HIV-1 of “self” recognition pathways that aid virus dissemination and might be the HIV-1 evasion strategy from DC intrinsic virus restriction factors.

Unlike other members of the Siglec family, which mostly interact with sialylated ligands in cis, because of the extensive cell surface sialylation that masks interactions in trans [24], CD169 is uniquely positioned to participate in both host-pathogen interactions and cell-to-cell interactions in trans, because of the 17 Ig-like domains that extends the receptor away from the cell surface [24]. In addition to mediating binding and endocytic entry of *Trypanosome cruzi* [66], *Campylobacter jejuni* [67] and porcine respiratory and reproductive syndrome virus, PRSSV [32], [68], results presented in this report suggest that capture of the simple retrovirus, MLV, is also dependent on CD169 and is facilitated via surface exposed terminal α2-3 sialoglycoconjugates. Interestingly, the capture of HIV-1 by CD169 results in virus localization in non-lysosomal compartments (Figure 3 and Supplementary Figure S4), nature of which still remain under debate [2], [6], [7], [8], [31], [69], [70]. While no endocytic or signaling motifs have been defined in the cytoplasmic tail of CD169 [25], distinct pathogen localization upon CD169 ligation might reflect the requirement for co-factor(s) driving trafficking of diverse pathogens to unique intracellular compartments.

Although CD169 is extremely well conserved in various mammalian species (from mouse to human), the physiologically-relevant sialylated ligand involved in CD169-dependent cell-to-cell adhesion and the biological role of CD169 in mammalian cells are not well defined. The extensive similarity between the murine and human CD169 proteins, especially in the extracellular domain, and our findings on MLV and HIV-1 interactions with human and murine CD169 (Figure 8), suggest that GSL-dependent capture of retrovirus particles is conserved across species. Furthermore, similar to the mechanism of HIV-1 trans infection, captured MLV particles were transferred to virus-susceptible cells in a CD169-dependent manner. While mouse myeloid DCs have previously been shown to capture and transfer HIV-1 particles to human CD4^+^ T cells [1], the virus attachment factor on murine myeloid DCs that mediates retroviral infection in trans has not been defined. Whether murine CD169 fulfills this function on murine myeloid DCs and whether CD169^+^ DCs can mediate dissemination of MLV in vivo remains to be determined. Interestingly, expression of murine CD169 has been reported predominantly on subsets of tissue resident and inflammatory macrophages lining the subcapsular sinus in peripheral lymph nodes and the spleen [24], with isolated descriptions of CD169 expression on migratory DCs in B cell follicles [71]. Thus, examination of the role of CD169 in MLV infection in vivo might provide valuable insights into the mechanisms of retroviral parasitization of antigen acquisition, antigen presentation and T cell stimulation functions of macrophages and DCs.

Human CD169 has a much broader expression pattern as it has been detected systematically on myeloid-derived cells. In addition to human DCs (described in this report, and [29], [49], [72]), monocyte-derived macrophages (MDMs) [73] and IFNα-exposed monocytes were also reported to express CD169 [26]. In contrast to our findings, CD169 on MDMs and IFNα-exposed monocytes was reported to bind soluble sialylated HIV-1 gp120 [73]. While theoretically possible, the limited number of Env trimers on virus particle surface [74] might preclude high efficiency interaction with CD169, considering that the binding affinity of Siglecs, including CD169, for their ligands is estimated to be in the micromolar (Kd) range. Interestingly, avidity of Siglec binding to ligands can be greatly enhanced upon challenge with geometrically arrayed, multivalent ligands [75], such as those when presented on virus capsid surface. Thus, in contrast to virus particle-associated Env, the high valency [18], [21] and, presumably, unique spacing of GM3 molecules on the surface of HIV-1 particles, might provide a multivalent scaffold for high efficiency binding to CD169^+^ DCs. We propose that there is a hierarchical order to ligand choice by DC-associated CD169 with virus capsid-associated GM3 providing the primary interaction interface, and that cell-intrinsic variability in surface localization and expression level of CD169 between DCs and macrophages might account for putative cell-type differences in HIV-1 particle-associated ligand specificity for CD169. While CD169 expression on macrophages can enhance productive infection by HIV-1 [73], whether CD169^+^ macrophages can also capture and trans infect HIV-1 in a GM3-dependent manner remains to be determined.

A cardinal feature of HIV-1 infections in vivo is high level of chronic immune activation, even observed in individuals on highly active antiretroviral therapy. Many factors have been attributed to cause HIV-associated immune activation including virus infection as well as translocation of microbial products from the intestinal lumen to the systemic circulation, and the release of high levels of pro-inflammatory cytokines, such as IL-6, TNFα and type I IFN [36]. Recent studies with the SIV – macaque model systems have demonstrated that intravaginal application of SIV_mac_ results in recruitment of plasmacytoid dendritic cells (pDCs) to the vaginal mucosa that upon detection of virus produce chemokines and type I IFN [35]. Furthermore, rapid and transient elevations in systemic IFNα have also been observed in acute HIV-1 infection [76]. While HIV-1 replication can be potently inhibited by type I IFNs at different steps of the viral life cycle in vitro [77], paradoxically, high tissue and plasma levels of IFNα as a consequence of overactive innate immune responses have been shown to correlate with rapid disease progression [36]. Intriguingly, exposure of HIV-1 infected cells to immune activation stimuli can result in increased incorporation of GM3 within HIV-1 particles derived from these cells [21]. Furthermore, recruitment of monocytes and myeloid DCs to peripheral tissues and subsequent IFNα-dependent differentiation and induction of CD169 on DCs might result in enhanced GM3-dependent virus interaction with CD169^+^ DCs, and access to DC-mediated trans infection pathway. Thus, utilization of the GM3 – CD169 axis of recognition is a subversion mechanism by which HIV utilizes DC functions to promote its replication.

## Materials and Methods

### Ethics Statement

This research has been determined to be exempt by the Institutional Review Board of the Boston University Medical Center since it does not meet the definition of human subjects research, since all human samples were collected in an anonymous fashion and no identifiable private information was collected.

### CD169 Cloning and Expression

Human CD169 was amplified from total RNA derived from IFN-α stimulated THP1 cells by overlap RT-PCR using the following primer sets: CD169 sense (TGTTAACATATGGCACAAGAACCTGCTATGG), CD169 internal-antisense (GCCTCAGTCAGGATGCGGCAGGCGTAAAGGGCAGCATCAGTT), CD169 internal-sense (AACTGATGCTGCCCTTTACGCCTGCCGCATCCTGACTGAGGC) and CD169 antisense (AGATATCTAGACAACACCACTGGTCAGCC) using Superscript III RT-PCR first strand synthesis kit (Invitrogen) and Phusion High Fidelity PCR (NEB). Mouse CD169 was amplified from total RNA derived from RAW264.7 mouse macrophage cell line by overlap RT-PCR using the following primer sets: muCD169 sense (ATAAGAATGCGGCCGCATGCACCTGGGCAC), muCD169 internal-antisense (AGCTCCGTTCAGGTACCAGGAGAAG), muCD169 internal-sense (AGCTCCTGACACTCGCTTCTCC) and muCD169 antisense (CGTCTAGACAGAGAGCAGCAACCACTTCC). Overlapping CD169 PCR fragments were first cloned into the pSC-B blunt cloning vector (Agilent Technologies) and then subsequently into the lentivector, pHIV-dTomato (Addgene) using EcoRI and XbaI restriction enzymes for human CD169 and NotI and XbaI restriction enzymes for mouse CD169 (pCD169-IRES-dTomato). Human CD169 was also cloned into a retroviral expression vector, LNCX (LNC-CD169). Point mutations in human CD169 orf, R116A (Arg→Ala) and R96A (Arg→Ala) were introduced by site-directed mutagenesis (Quikchange; Agilent Technologies). All clones were verified by sequencing. Stable expression of CD169 in Raji B cell line was accomplished by transduction either with VSV-G pseudotyped pCD169-IRES-dTomato lentivector and cell sorting for dTomato expression, or LNC-CD169 retroviral vector followed by G418 selection. CD169 was transiently expressed in HEK293T cells by calcium phosphate-mediated transfection, and protein expression confirmed by western blot analysis and/or FACS.

### Cells

Human cell lines, HEK293T, Raji and TZM-Bl, have been described before [13], [14], [21], [47]. Rat-2, a rat fibroblast cell line, was obtained from ATCC. PBMC, isolated from leukupaks of healthy donors, were activated with 2% phytohemagglutinin (PHA) (Invitrogen) and 50 U/ml rhIL-2 (Roche, NIAID ARRRP) in RPMI/10%FBS. Primary monocyte derived DCs were differentiated from CD14^+^ peripheral blood monocytes, as described previously [14]. Immature DCs were matured by incubation with either LPS (100 ng/ml), poly(I:C) (25 µg/ml), or Pam_3_CysK_4_ (100 ng/ml) for 2 days. Alternatively, DCs were matured by addition of cytokines, IFNα (1000 U/ml) or TNFα (10 ng/ml) for 48 h. To generate IFN-DCs, CD14^+^ monocytes were cultured in the presence of GM-CSF (1,400 U/ml) and IFNα (1000 U/ml) for 3 days [42]. Autologous activated CD4^+^ T cells were derived as described previously [21]. Blood myeloid DCs were positively isolated from PBMCs using BDCA-1 conjugated magnetic beads (Miltenyi) and cultured in RPMI/10%FBS containing 20 U/ml GM-CSF or stimulated with 1000 U/ml IFNα for 48 h prior to use. The following antibodies (all from Becton Dickinson) were used for DC immunophenotyping analysis: PE-conjugated CD14, CD11c, HLA-DR, CD83, CD4, and CCR5; and FITC-conjugated α-CD86 and α-DC-SIGN; Alexa488- or Alex647-conjugated α-CD169 (AbD Serotec), and isotype controls. FACS analysis was performed as described before [21].

### Virus and VLPs

CXCR4-tropic HIV/Lai, CCR5-tropic HIV/Lai-Bal and luciferase expressing HIV/Lai-Bal-luc provirus clones have been described previously [78]. To generate HIV/Lai-iGFP and HIV/Lai-iGFPΔenv molecular clones the eGFP fragment flanked by HIV-1 protease cleavage sites from HIV/NL43-iGFP (gift of Dr. Benjamin Chen, Mt Sinai School of Medicine) was exchanged into HIV/Lai. HIV Gag-eGFP and HIV Gag-mCherry expression plasmids that express codon-optimized Gag-eGFP and Gag-mCherry fusion proteins have been described previously [7]. MLV Gag-YFP expression plasmid has been described previously [79]. HIV Gag-eGFP and MLV Gag-YFP VLPs and infectious viruses were either derived via calcium phosphate mediated transfection of HEK293T cells, as described previously [21]. Luciferase expressing, single cycle of replication competent MLV particles (MLV-E/luc) were derived via co-transfection of HEK293T cells with LNC-luc (luciferase expressing retroviral expression vector) and pCL-Eco (MLV packaging vector that expresses MLV Gag, Pol and ecotropic Env glycoproteins; Imgenex) plasmids. To derive GSL depleted virus particles, HEK293T cells were pre-treated with 10 µM PDMP (Calbiochem) for 24 h prior to transfection, and cells were maintained in the presence of PDMP (10 µM) during the course of transfection [14]. To produce PBMC-derived viruses, cells were infected with VSV-G double Env pseudotyped HIV/Lai-Bal virus particles in the presence or absence of PDMP (10 µM; cells pre-treated for 2 days), and the drug concentration was maintained for the duration of the infection. Cell-free supernatants were harvested 3 and 6 days post infection and stored at −80°C until further use. To determine the infectious titer of the PBMC and HEK293T cell derived viruses, 10^4^ TZM-Bl cells were infected with limiting dilutions of virus stocks. Cells were lysed 48 h post infection, and cell lysates were analyzed for luciferase activity using a commercially available kit (Promega).

### GSL and Env Content of Virus Particles

Production and quantification of HIV Gag-eGFP or MLV Gag-YFP particles from transiently transfected HEK293T cells in the presence or absence of PDMP was confirmed by quantitative LICOR-western blot analysis using goat α-GFP polyclonal antibody. To quantify virus particle incorporation of GSLs, virus particles (100 ng Gag-eGFP or MLV Gag-YFP) derived from untreated or PDMP-treated HEK293T cells were incubated with 2 µg cholera toxin subunit B (CtxB)-biotin (Sigma), coupled to 25 µl streptavidin coated 2.8 µM dynabeads (Invitrogen) in 1% casein/PBS (Pierce) at RT for 1 h with rotation, washed twice with 0.1% casein/PBS and beads-associated virions lysed with SDS-sample buffer. The amount of immunoprecipitated Gag-eGFP or MLV Gag-YFP was determined using quantitative western blot analysis with recombinant GFP (Roche) as standards. Membranes were probed with goat α-GFP (Novus Biologicals) followed by donkey α-goat-IgG-IRDye 800CW (Li-Cor, Lincoln, Nebraska). To measure GM3 incorporation in HIV/Lai-Bal virus particles, virions were stained with α-GM3 mAb [80] and Alexa594-conjugated goat α-mouse IgG (Invitrogen). Quantification of GM3 staining in virus particles derived from untreated or PDMP (10 µM)-treated virus producer cells was determined, as described previously [21]. The p24^gag^ content and gp120 incorporation in HIV-1 particles derived from HEK293T or PBMC was determined using mouse α-gag mAb (Clone p24-2, NIH AIDS Research and Reference Reagent Program, contributed by Dr. Michael Malim) and rabbit α-gp120 polyclonal antibody (gift of Dr. Nancy Haigwood) and staining visualized by goat α-mouse-IRDye 680CW or goat α-rabbit-IRDye 800CW (Licor), respectively. Membranes were imaged with an Odyssey scanner (Licor) and the amount of gp120 or p24^gag^ or Gag-eGFP in the samples was quantified using recombinant HIV-1 Bal gp120 (ARRRP) or HIV-1 IIIB p24 (ABL, Rockville, MD) as standards, respectively.

### Lentivector-shRNA Production and DC Transductions

Lentivirus particles expressing CD169, DC-SIGN or scrambled shRNAs were produced via transient transfections of HEK293T cells with lentiviral vectors (Pierce, Rockford IL) and packaging plasmids psPAX2 and pMD2G (Addgene). Immature DCs, 2 days post initiation of differentiation from CD14^+^ monocytes, were infected with lentiviral vectors by spin-inoculation in the presence of SIV Vpx containing VLPs, as described previously [21]. Cells were washed with PBS 24 h post transduction and cultured for 48 h. DCs were stimulated with 100 ng/ml LPS and vector-transduced cells were selected with 2 µg/ml puromycin for 48 h prior to use. To assess expression of CD169 and DC-SIGN on shRN-transduced cells, membranes were probed with a mouse α-CD169 antibody (7D2, Novus Biologicals) and a mouse α-DC-SIGN antibody (DC28, NIH AIDS Research and Reference Reagent Program Catalog #5443; contributed by Dr. R.W. Doms), respectively. As loading controls, either actin or HSP70 was detected using rabbit α-actin (SIGMA, St. Louis, MO) or mouse α-HSP70 (Stressgen, Victoria, BC) antibodies, respectively.

### Virus Capture and Transfer Assays

Infectious virus and HIV Gag-eGFP and MLV Gag-YFP VLP capture assays and HIV and MLV trans infection assays were performed as described previously [21]. Briefly, cells (1×10^5^) were incubated with virus (20 ng p24^gag^ unless noted otherwise) or VLPs (2 ng p24^gag^) for 2 hr at 37°C in raw RPMI media. Cells were washed 3× with 1×PBS and analyzed for capture using either p24^gag^ ELISA or FACS analysis. Virus capture was quantified by ng/ml of p24^gag^ associated with cell lysates using a p24^gag^ ELISA that has been described previously [14]. Capture of fluorescent VLPs was detected by FACSCalibur and quantified for percent positive cells. For transfer assays, cells (1×10^5^) were incubated with virus particles at 37°C for 2 hr, washed 3× with 1×PBS and co-cultured with CD4^+^ T cells (for HIV-1 transfer) or Rat-2 cells (for MLV transfer) at a 1∶1 cell ratio in complete RPMI media. To competitively inhibit virus binding, DCs (1.0×10^5^ cells) were pre-incubated with 20 µg/ml final concentration of α-CD169 (7D2, Novus Biologicals, Littleton CO), α-DC-SIGN (14EG7, NIH AIDS Research and Reference Reagent Program Catalog #11423), murine IgG2b or IgG1 isotype controls (eBioscience) for 30 minutes at room temperature prior to virus exposure. To determine the effect of cell surface-associated sialic acid residues on VLP capture, mature DCs were treated with neuraminidase that efficiently cleaves α2-3, α2-6, and α2-8 sialic acids (NEB) for 1 h at 37°C prior to challenge with HIV Gag-eGFP VLPs, as described previously [21]. Fluorescent unilamellar liposomes containing GSLs were produced as described previously [21]. For liposome capture and virus inhibition assays, sodium azide-treated cells (10^5^ per well) were incubated with liposomes for 45 min at 37°C, and cells either fixed and processed for FACS analysis, or challenged with HIV/Lai-iGFPΔenv virus particles (20 ng p24^gag^) for 1 h at 37°C. Virus capture was determined by measuring cell-associated p24^gag^ by ELISA.

### QFACS Analysis

To determine the number of CD169 and DC-SIGN molecules on DCs, antibody-binding sites (ABS) were quantified using a microbeads kit (Bangs Laboratories). Beads (10^5^) and cells were stained with saturating amounts of FITC-conjugated α-DC-SIGN or IgG2b isotype control (both from BD), or Alexa647-conjugated α-CD169 or IgG1 isotype control (both from AbD Serotec) for 30 minutes on ice. Stained populations were detected using a LSRII (BD), and the data analyzed using FlowJo software (Treestar). For analysis, auto-fluorescence signals from unstained samples were subtracted from GMFIs of stained samples, and ABS of CD169, DC-SIGN or isotype controls on DCs were calculated from standard curves generated from stained beads.

### Co-localization Analysis

To visualize virus localization, mature DCs (1.5×10^5^) were incubated with 10 ng Gag-mCherry VLPs or 500 ng of Lai-iGFP virus particles at 37°C for 10 minutes or 2 h. For conjugate analyses, DCs were incubated with virus particles for 1 h, washed and co-cultured for an additional 1 h with autologous CD4^+^ T cells (1∶1 DC∶T ratio) pre-labeled with 5 µM CMAC (CellTracker Blue, Invitrogen). Cells were fixed with 1% PFA, blocked with 20% NHS, and probed with Alexa-488 conjugated α-CD169 or stained with α-CD45, α-CD9, α-CD81, α-EEA-1, α-Lamp1 mAbs (all antibodies from BD) or isotype controls and detected with Alexa488-conjugated 2°Abs (Invitrogen). Confocal Z-stacks were captured with an Olympus DSU spinning disk at 60× and processed with ImageJ software. For visualization, images were deconvolved, background subtracted, and flattened for maximum intensity.

### Statistical Analysis

All virus capture and transfer values and FACS MFIs are expressed as means ± SD. Statistical significance was determined by Student's one-tailed *t* test, and significant P values (<0.05) are indicated on the figures. To quantify co-localization analysis, single z-stack slices from 10 independent cells were assessed using Mander's coefficient and the average ratio of red (Gag-mCherry) associated with green (CD169/isotype control/CD45) +/− SD is reported.

## Supporting Information

Figure S1 **GSL-dependent HIV-1 capture mechanism is protease sensitive and is specific to myeloid cells.** A. Gag-eGFP VLP capture assays were performed on mature DCs untreated (mock) or treated with 2 mg/ml pronase prior to HIV Gag-eGFP VLP exposure. B. HIV Gag-eGFP VLP capture by cells untreated (mock) or treated with IFNα (1000 U/ml) for 48 h prior to VLP exposure. Reported data in panels A and B are relative capture normalized to mock treated cells (from 4 independent experiments; mean +/−SD).
(TIF)

Figure S2 **Selective depletion of HIV-1 attachment factor expression in dendritic cells.** Immature DCs transduced with shRNAs expressing lentivectors targeting DC-SIGN, CD169, or scrambled sequence were stimulated with LPS (100 ng/ml) for 48 h and assayed for cell surface expression by FACS (A, B) or total cellular expression by western blot analysis (C, D) of CD169 (A, C) or DC-SIGN (B, D). Cell surface expression of CD169 (A) or DC-SIGN (B) is reported as relative MFI expression to that of cells transduced with lentivectors expressing scrambled shRNA, and is the average of three independent experiments (mean ± SD).
(TIF)

Figure S3 **CD169 is the sole SIGLEC family member responsible for HIV-1 capture by dendritic cells.** Mature DCs, left untreated or pre-treated with neuraminidase, were incubated with 1 µg of antibody directed against CD169 (Siglec-1), Siglec-7, or Siglec-9. Capture assays with HIV Gag-eGFP VLPs were performed in duplicate on mature DCs from two independent donors, and the average Gag-eGFP VLP capture +/− SD is reported.
(TIF)

Figure S4 **HIV-1 particles captured by mature DCs are co-localized with CD169.** (A) Co-localization of HIV/Lai-iGFP (green) with CD169 (red) on mature DC surface within 10 minutes of virus exposure, (B) and in peripheral polarized compartment upon 120 minutes of virus exposure. (C–G) Mature DCs incubated with Gag-mCherry VLP (red) for <10 minutes were probed for cell surface (CD9) and endosomal markers (EEA1 and LAMP1). Staining of cellular markers was visualized by Alexa488-conjugated secondary antibodies (green); representative images are shown for staining with (C) CD9, (D) EEA1 and (E) LAMP1. Lack of co-localization between CD45 (green) and HIV Gag-mCherry VLP in mature DCs after 10 min (F) or 120 min (G) post virus exposure.
(TIF)

Figure S5 **Differential expression of CD169 and DC-SIGN on IFN-α and IL4 differentiated DCs.** Immunophenotypic characterization of IFN-DCs (GM-CSF + IFNα 3 days post initiation of differentiation) (A) and IL4-DCs (GM-CSF + IL-4, 3 days post-initiation of differentiation) (B) was determined by FACS analysis. The red histograms represent staining with the isotype control antibody and the blue histograms represent staining for antibodies to the specific cell surface markers.
(TIF)

Figure S6 **HIV Gag-eGFP VLPs produced from PDMP-treated HEK293T cells are depleted in GSLs.** The model depicts the simplified GSL biosynthesis pathway, and the enzymatic step (synthesis of glucosylceramide, catalyzed by the enzyme, glucosylceramide synthase) inhibited by the cationic lipid, PDMP (A). The amount of HIV Gag-eGFP VLPs produced from transient transfection of HEK293T cells in the presence or absence (NT) of PDMP (10 µM), is quantified by quantitative LICOR-western blot analysis (B) using a α-GFP polyclonal antibody. The relative incorporation of GSLs in VLPs derived from untreated (NT) or PDMP-treated HEK293T cells were determined by immunoprecipitation with biotin-conjugated CtxB and streptavidin-dynabeads. Quantification of the immunoprecipitated virus particles was enabled by quantitative western blot analysis using a α-GFP polyclonal antibody (C).
(TIF)

Figure S7 **Depletion of GSLs from HEK293T or PBMC-derived HIV-1 particles attenuates virus capture by IFN-DCs.** A. HIV-1 Env (gp120) and p24^gag^ content of HIV/Lai-Bal virus particles derived from HEK293T or PBMCs in the absence (NT) or presence of PDMP (10 µM), was determined by quantitative LICOR-western blot analysis using α-gp120 and α-p24^gag^ primary antibodies and IR680 and IR800-conjugated secondary antibodies, respectively. Virions (HIV/Lai-Bal) derived from untreated (B) or PDMP-treated (C) PBMCs were labeled for p24^gag^ (green) and GM3 (red). Representative fields are shown and the average mean fluorescence intensity of GM3 normalized to p24^gag^ ± SD is reported for HEK293T (D) and PBMC-derived (E) virus stocks. F. Infectivity of HIV/Lai-Bal derived from PBMCs in the absence (NT) or presence of PDMP (10 µM) was determined on TZM-bl reporter cells. G. Capture assays with IFN-DCs and IL4-DCs were performed with PBMC-derived HIV/Lai-Bal (±PDMP) and cell-associated p24^gag^ content determined by ELISA. Data reported is average of three independent experiments, +/− SD.
(TIF)

Figure S8 **Mutation of the sialic acid recognition motif in CD169 abrogates HIV-1 capture.** Expression of CD169 or mutants, R96A and R116A, in transiently transfected HEK293T cells was determined by western blot analysis (A). The percentage of CD169 (or mutant) positive cells capturing HIV Gag-eGFP VLPs was determined by FACS analysis (B). The data reported is the average of two independent experiments performed in triplicate (mean ± SD).
(TIF)

Figure S9 **Characterization of MLV Gag-YFP VLPs.** (A) The amount of MLV Gag-YFP VLPs produced from transient transfection of untreated (NT) or PDMP (10 µM) treated HEK293T cells was quantified by quantitative LICOR-western blot analysis using an α-GFP polyclonal antibody and IRDye 800CW-conjugated donkey α-goat-IgG secondary antibody (B) and α-MLV Gag monoclonal antibody. B. The relative incorporation of GSLs in MLV Gag-YFP VLPs derived from untreated (NT) or PDMP-treated HEK293T cells were determined by immunoprecipitation with biotin-conjugated CtxB and streptavidin-dynabeads. Quantification of the immunoprecipitated virus particles was enabled by quantitative western blot analysis using an anti-GFP polyclonal antibody. C. Capture of MLV Gag-YFP VLPs (±PDMP) by Raji (C) and Raji/CD169 cells (D) was determined by measuring the percentage of YFP positive cells by FACS. The data reported is representative of two independent experiments performed in duplicate.
(TIF)

## References

[1] Cameron PU, Freudenthal PS, Barker JM, Gezelter S, Inaba K, et al (1992) Dendritic cells exposed to human immunodeficiency virus type-1 transmit a vigorous cytopathic infection to CD4+ T cells. Science 257: 383–387. 1352913 10.1126/science.1352913

[2] McDonald D, Wu L, Bohks SM, KewalRamani VN, Unutmaz D, et al (2003) Recruitment of HIV and its receptors to dendritic cell-T cell junctions. Science 300: 1295–1297. 12730499 10.1126/science.1084238

[3] Wu L, KewalRamani VN (2006) Dendritic-cell interactions with HIV: infection and viral dissemination. Nat Rev Immunol 6: 859–868. 17063186 10.1038/nri1960 PMC1796806

[4] Turville SG, Cameron PU, Handley A, Lin G, Pohlmann S, et al (2002) Diversity of receptors binding HIV on dendritic cell subsets. Nat Immunol 3: 975–983. 12352970 10.1038/ni841

[5] Geijtenbeek TB, Kwon DS, Torensma R, van Vliet SJ, van Duijnhoven GC, et al (2000) DC-SIGN, a dendritic cell-specific HIV-1-binding protein that enhances trans-infection of T cells. Cell 100: 587–597. 10721995 10.1016/s0092-8674(00)80694-7

[6] Garcia E, Pion M, Pelchen-Matthews A, Collinson L, Arrighi JF, et al (2005) HIV-1 trafficking to the dendritic cell-T-cell infectious synapse uses a pathway of tetraspanin sorting to the immunological synapse. Traffic 6: 488–501. 15882445 10.1111/j.1600-0854.2005.00293.x

[7] Izquierdo-Useros N, Naranjo-Gomez M, Archer J, Hatch SC, Erkizia I, et al (2009) Capture and transfer of HIV-1 particles by mature dendritic cells converges with the exosome-dissemination pathway. Blood 113: 2732–2741. 18945959 10.1182/blood-2008-05-158642 PMC2661860

[8] Yu HJ, Reuter MA, McDonald D (2008) HIV traffics through a specialized, surface-accessible intracellular compartment during trans-infection of T cells by mature dendritic cells. PLoS Pathog 4: e1000134. 18725936 10.1371/journal.ppat.1000134 PMC2515344

[9] Engering A, Geijtenbeek TB, van Kooyk Y (2002) Immune escape through C-type lectins on dendritic cells. Trends Immunol 23: 480–485. 12297419 10.1016/s1471-4906(02)02296-2

[10] Kwon DS, Gregorio G, Bitton N, Hendrickson WA, Littman DR (2002) DC-SIGN-mediated internalization of HIV is required for trans-enhancement of T cell infection. Immunity 16: 135–144. 11825572 10.1016/s1074-7613(02)00259-5

[11] Boggiano C, Manel N, Littman DR (2007) Dendritic cell-mediated trans-enhancement of human immunodeficiency virus type 1 infectivity is independent of DC-SIGN. J Virol 81: 2519–2523. 17182696 10.1128/JVI.01661-06 PMC1865951

[12] Granelli-Piperno A, Pritsker A, Pack M, Shimeliovich I, Arrighi JF, et al (2005) Dendritic cell-specific intercellular adhesion molecule 3-grabbing nonintegrin/CD209 is abundant on macrophages in the normal human lymph node and is not required for dendritic cell stimulation of the mixed leukocyte reaction. J Immunol 175: 4265–4273. 16177066 10.4049/jimmunol.175.7.4265 PMC1378112

[13] Gummuluru S, Rogel M, Stamatatos L, Emerman M (2003) Binding of human immunodeficiency virus type 1 to immature dendritic cells can occur independently of DC-SIGN and mannose binding C-type lectin receptors via a cholesterol-dependent pathway. J Virol 77: 12865–12874. 14610207 10.1128/JVI.77.23.12865-12874.2003 PMC262553

[14] Hatch SC, Archer J, Gummuluru S (2009) Glycosphingolipid composition of human immunodeficiency virus type 1 (HIV-1) particles is a crucial determinant for dendritic cell-mediated HIV-1 trans-infection. J Virol 83: 3496–3506. 19193785 10.1128/JVI.02249-08 PMC2663285

[15] Wang JH, Janas AM, Olson WJ, Wu L (2007) Functionally distinct transmission of human immunodeficiency virus type 1 mediated by immature and mature dendritic cells. J Virol 81: 8933–8943. 17567699 10.1128/JVI.00878-07 PMC1951429

[16] Nguyen DH, Hildreth JE (2000) Evidence for budding of human immunodeficiency virus type 1 selectively from glycolipid-enriched membrane lipid rafts. J Virol 74: 3264–3272. 10708443 10.1128/jvi.74.7.3264-3272.2000 PMC111827

[17] Ono A, Freed EO (2001) Plasma membrane rafts play a critical role in HIV-1 assembly and release. Proc Natl Acad Sci U S A 98: 13925–13930. 11717449 10.1073/pnas.241320298 PMC61143

[18] Chan R, Uchil PD, Jin J, Shui G, Ott DE, et al (2008) Retroviruses human immunodeficiency virus and murine leukemia virus are enriched in phosphoinositides. J Virol 82: 11228–11238. 18799574 10.1128/JVI.00981-08 PMC2573248

[19] Chertova E, Chertov O, Coren LV, Roser JD, Trubey CM, et al (2006) Proteomic and biochemical analysis of purified human immunodeficiency virus type 1 produced from infected monocyte-derived macrophages. J Virol 80: 9039–9052. 16940516 10.1128/JVI.01013-06 PMC1563931

[20] Brugger B, Glass B, Haberkant P, Leibrecht I, Wieland FT, et al (2006) The HIV lipidome: a raft with an unusual composition. Proc Natl Acad Sci U S A 103: 2641–2646. 16481622 10.1073/pnas.0511136103 PMC1413831

[21] Puryear WB, Yu X, Ramirez NP, Reinhard BM, Gummuluru S (2012) HIV-1 incorporation of host-cell-derived glycosphingolipid GM3 allows for capture by mature dendritic cells. Proc Natl Acad Sci U S A 109: 7475–7480. 22529395 10.1073/pnas.1201104109 PMC3358844

[22] Izquierdo-Useros N, Lorizate M, Contreras FX, Rodriguez-Plata MT, Glass B, et al (2012) Sialyllactose in viral membrane gangliosides is a novel molecular recognition pattern for mature dendritic cell capture of HIV-1. PLoS Biol 10: e1001315. 22545022 10.1371/journal.pbio.1001315 PMC3335875

[23] Barton GM, Kagan JC (2009) A cell biological view of Toll-like receptor function: regulation through compartmentalization. Nat Rev Immunol 9: 535–542. 19556980 10.1038/nri2587 PMC3934928

[24] Crocker PR, Paulson JC, Varki A (2007) Siglecs and their roles in the immune system. Nat Rev Immunol 7: 255–266. 17380156 10.1038/nri2056

[25] Hartnell A, Steel J, Turley H, Jones M, Jackson DG, et al (2001) Characterization of human sialoadhesin, a sialic acid binding receptor expressed by resident and inflammatory macrophage populations. Blood 97: 288–296. 11133773 10.1182/blood.v97.1.288

[26] Rempel H, Calosing C, Sun B, Pulliam L (2008) Sialoadhesin expressed on IFN-induced monocytes binds HIV-1 and enhances infectivity. PLoS One 3: e1967. 18414664 10.1371/journal.pone.0001967 PMC2288672

[27] York MR, Nagai T, Mangini AJ, Lemaire R, van Seventer JM, et al (2007) A macrophage marker, Siglec-1, is increased on circulating monocytes in patients with systemic sclerosis and induced by type I interferons and toll-like receptor agonists. Arthritis Rheum 56: 1010–1020. 17328080 10.1002/art.22382

[28] Biesen R, Demir C, Barkhudarova F, Grun JR, Steinbrich-Zollner M, et al (2008) Sialic acid-binding Ig-like lectin 1 expression in inflammatory and resident monocytes is a potential biomarker for monitoring disease activity and success of therapy in systemic lupus erythematosus. Arthritis Rheum 58: 1136–1145. 18383365 10.1002/art.23404

[29] Kirchberger S, Majdic O, Steinberger P, Bluml S, Pfistershammer K, et al (2005) Human rhinoviruses inhibit the accessory function of dendritic cells by inducing sialoadhesin and B7-H1 expression. J Immunol 175: 1145–1152. 16002716 10.4049/jimmunol.175.2.1145

[30] Baribaud F, Pohlmann S, Leslie G, Mortari F, Doms RW (2002) Quantitative expression and virus transmission analysis of DC-SIGN on monocyte-derived dendritic cells. J Virol 76: 9135–9142. 12186897 10.1128/JVI.76.18.9135-9142.2002 PMC136426

[31] Felts RL, Narayan K, Estes JD, Shi D, Trubey CM, et al (2010) 3D visualization of HIV transfer at the virological synapse between dendritic cells and T cells. Proc Natl Acad Sci U S A 107: 13336–13341. 20624966 10.1073/pnas.1003040107 PMC2922156

[32] Delputte PL, Van Gorp H, Favoreel HW, Hoebeke I, Delrue I, et al (2011) Porcine sialoadhesin (CD169/Siglec-1) is an endocytic receptor that allows targeted delivery of toxins and antigens to macrophages. PLoS One 6: e16827. 21359217 10.1371/journal.pone.0016827 PMC3040196

[33] Sallusto F, Lanzavecchia A (1994) Efficient presentation of soluble antigen by cultured human dendritic cells is maintained by granulocyte/macrophage colony-stimulating factor plus interleukin 4 and downregulated by tumor necrosis factor alpha. J Exp Med 179: 1109–1118. 8145033 10.1084/jem.179.4.1109 PMC2191432

[34] Nelms K, Keegan AD, Zamorano J, Ryan JJ, Paul WE (1999) The IL-4 receptor: signaling mechanisms and biologic functions. Annu Rev Immunol 17: 701–738. 10358772 10.1146/annurev.immunol.17.1.701

[35] Li Q, Estes JD, Schlievert PM, Duan L, Brosnahan AJ, et al (2009) Glycerol monolaurate prevents mucosal SIV transmission. Nature 458: 1034–1038. 19262509 10.1038/nature07831 PMC2785041

[36] Sandler NG, Douek DC (2012) Microbial translocation in HIV infection: causes, consequences and treatment opportunities. Nat Rev Microbiol 10: 655–666. 22886237 10.1038/nrmicro2848

[37] Shi C, Pamer EG (2011) Monocyte recruitment during infection and inflammation. Nat Rev Immunol 11: 762–774. 21984070 10.1038/nri3070 PMC3947780

[38] Krutzik SR, Tan B, Li H, Ochoa MT, Liu PT, et al (2005) TLR activation triggers the rapid differentiation of monocytes into macrophages and dendritic cells. Nat Med 11: 653–660. 15880118 10.1038/nm1246 PMC1409736

[39] Naik SH, Metcalf D, van Nieuwenhuijze A, Wicks I, Wu L, et al (2006) Intrasplenic steady-state dendritic cell precursors that are distinct from monocytes. Nat Immunol 7: 663–671. 16680143 10.1038/ni1340

[40] Randolph GJ, Beaulieu S, Lebecque S, Steinman RM, Muller WA (1998) Differentiation of monocytes into dendritic cells in a model of transendothelial trafficking. Science 282: 480–483. 9774276 10.1126/science.282.5388.480

[41] Paquette RL, Hsu NC, Kiertscher SM, Park AN, Tran L, et al (1998) Interferon-alpha and granulocyte-macrophage colony-stimulating factor differentiate peripheral blood monocytes into potent antigen-presenting cells. J Leukoc Biol 64: 358–367. 9738663 10.1002/jlb.64.3.358

[42] Santini SM, Lapenta C, Logozzi M, Parlato S, Spada M, et al (2000) Type I interferon as a powerful adjuvant for monocyte-derived dendritic cell development and activity in vitro and in Hu-PBL-SCID mice. J Exp Med 191: 1777–1788. 10811870 10.1084/jem.191.10.1777 PMC2193160

[43] Lin G, Simmons G, Pohlmann S, Baribaud F, Ni H, et al (2003) Differential N-linked glycosylation of human immunodeficiency virus and Ebola virus envelope glycoproteins modulates interactions with DC-SIGN and DC-SIGNR. J Virol 77: 1337–1346. 12502850 10.1128/JVI.77.2.1337-1346.2003 PMC140807

[44] de Witte L, Bobardt M, Chatterji U, Degeest G, David G, et al (2007) Syndecan-3 is a dendritic cell-specific attachment receptor for HIV-1. Proc Natl Acad Sci U S A 104: 19464–19469. 18040049 10.1073/pnas.0703747104 PMC2148312

[45] Izquierdo-Useros N, Blanco J, Erkizia I, Fernandez-Figueras MT, Borras FE, et al (2007) Maturation of blood-derived dendritic cells enhances human immunodeficiency virus type 1 capture and transmission. J Virol 81: 7559–7570. 17475656 10.1128/JVI.02572-06 PMC1933337

[46] Turville SG, Arthos J, MacDonald K, Lynch G, Naif H, et al (2001) HIV gp120 receptors on human dendritic cells. Blood 98: 2482–2488. 11588046 10.1182/blood.v98.8.2482

[47] Wu L, Martin TD, Carrington M, KewalRamani VN (2004) Raji B cells, misidentified as THP-1 cells, stimulate DC-SIGN-mediated HIV transmission. Virology 318: 17–23. 14972530 10.1016/j.virol.2003.09.028

[48] Vinson M, van der Merwe PA, Kelm S, May A, Jones EY, et al (1996) Characterization of the sialic acid-binding site in sialoadhesin by site-directed mutagenesis. J Biol Chem 271: 9267–9272. 8621587 10.1074/jbc.271.16.9267

[49] Izquierdo-Useros N, Lorizate M, Puertas MC, Rodriguez-Plata MT, Zangger N, et al (2012) Siglec-1 Is a Novel Dendritic Cell Receptor That Mediates HIV-1 Trans-Infection Through Recognition of Viral Membrane Gangliosides. PLoS Biol 10: e1001448. 23271952 10.1371/journal.pbio.1001448 PMC3525531

[50] Turville SG, Santos JJ, Frank I, Cameron PU, Wilkinson J, et al (2004) Immunodeficiency virus uptake, turnover, and 2-phase transfer in human dendritic cells. Blood 103: 2170–2179. 14630806 10.1182/blood-2003-09-3129

[51] Burleigh L, Lozach PY, Schiffer C, Staropoli I, Pezo V, et al (2006) Infection of dendritic cells (DCs), not DC-SIGN-mediated internalization of human immunodeficiency virus, is required for long-term transfer of virus to T cells. J Virol 80: 2949–2957. 16501104 10.1128/JVI.80.6.2949-2957.2006 PMC1395470

[52] Moris A, Nobile C, Buseyne F, Porrot F, Abastado JP, et al (2004) DC-SIGN promotes exogenous MHC-I-restricted HIV-1 antigen presentation. Blood 103: 2648–2654. 14576049 10.1182/blood-2003-07-2532

[53] Moris A, Pajot A, Blanchet F, Guivel-Benhassine F, Salcedo M, et al (2006) Dendritic cells and HIV-specific CD4+ T cells: HIV antigen presentation, T-cell activation, and viral transfer. Blood 108: 1643–1651. 16675708 10.1182/blood-2006-02-006361

[54] Arrighi JF, Pion M, Garcia E, Escola JM, van Kooyk Y, et al (2004) DC-SIGN-mediated infectious synapse formation enhances X4 HIV-1 transmission from dendritic cells to T cells. J Exp Med 200: 1279–1288. 15545354 10.1084/jem.20041356 PMC2211914

[55] Gummuluru S, KewalRamani VN, Emerman M (2002) Dendritic cell-mediated viral transfer to T cells is required for human immunodeficiency virus type 1 persistence in the face of rapid cell turnover. J Virol 76: 10692–10701. 12368311 10.1128/JVI.76.21.10692-10701.2002 PMC136613

[56] Wu L, Martin TD, Vazeux R, Unutmaz D, KewalRamani VN (2002) Functional evaluation of DC-SIGN monoclonal antibodies reveals DC-SIGN interactions with ICAM-3 do not promote human immunodeficiency virus type 1 transmission. J Virol 76: 5905–5914. 12021323 10.1128/JVI.76.12.5905-5914.2002 PMC136240

[57] Lee B, Leslie G, Soilleux E, O'Doherty U, Baik S, et al (2001) cis Expression of DC-SIGN allows for more efficient entry of human and simian immunodeficiency viruses via CD4 and a coreceptor. J Virol 75: 12028–12038. 11711593 10.1128/JVI.75.24.12028-12038.2001 PMC116098

[58] Nobile C, Petit C, Moris A, Skrabal K, Abastado JP, et al (2005) Covert human immunodeficiency virus replication in dendritic cells and in DC-SIGN-expressing cells promotes long-term transmission to lymphocytes. J Virol 79: 5386–5399. 15827153 10.1128/JVI.79.9.5386-5399.2005 PMC1082762

[59] Wang JH, Janas AM, Olson WJ, KewalRamani VN, Wu L (2007) CD4 coexpression regulates DC-SIGN-mediated transmission of human immunodeficiency virus type 1. J Virol 81: 2497–2507. 17151103 10.1128/JVI.01970-06 PMC1865928

[60] Hijazi K, Wang Y, Scala C, Jeffs S, Longstaff C, et al (2011) DC-SIGN increases the affinity of HIV-1 envelope glycoprotein interaction with CD4. PLoS One 6: e28307. 22163292 10.1371/journal.pone.0028307 PMC3233575

[61] Cavrois M, Neidleman J, Kreisberg JF, Fenard D, Callebaut C, et al (2006) Human immunodeficiency virus fusion to dendritic cells declines as cells mature. J Virol 80: 1992–1999. 16439555 10.1128/JVI.80.4.1992-1999.2006 PMC1367165

[62] Hrecka K, Hao C, Gierszewska M, Swanson SK, Kesik-Brodacka M, et al (2011) Vpx relieves inhibition of HIV-1 infection of macrophages mediated by the SAMHD1 protein. Nature 474: 658–661. 21720370 10.1038/nature10195 PMC3179858

[63] Laguette N, Sobhian B, Casartelli N, Ringeard M, Chable-Bessia C, et al (2011) SAMHD1 is the dendritic- and myeloid-cell-specific HIV-1 restriction factor counteracted by Vpx. Nature 474: 654–657. 21613998 10.1038/nature10117 PMC3595993

[64] Manel N, Hogstad B, Wang Y, Levy DE, Unutmaz D, et al (2010) A cryptic sensor for HIV-1 activates antiviral innate immunity in dendritic cells. Nature 467: 214–217. 20829794 10.1038/nature09337 PMC3051279

[65] Wilson SJ, Schoggins JW, Zang T, Kutluay SB, Jouvenet N, et al (2012) Inhibition of HIV-1 particle assembly by 2′,3′-cyclic nucleotide 3′-phosphodiesterase. Cell host & microbe 12: 585–597. 23084924 10.1016/j.chom.2012.08.012 PMC3498451

[66] Monteiro VG, Lobato CS, Silva AR, Medina DV, de Oliveira MA, et al (2005) Increased association of Trypanosoma cruzi with sialoadhesin positive mice macrophages. Parasitol Res 97: 380–385. 16151743 10.1007/s00436-005-1460-1

[67] Klaas M, Oetke C, Lewis LE, Erwig LP, Heikema AP, et al (2012) Sialoadhesin Promotes Rapid Proinflammatory and Type I IFN Responses to a Sialylated Pathogen, Campylobacter jejuni. J Immunol 189: 2414–2422. 22851711 10.4049/jimmunol.1200776 PMC3442253

[68] Vanderheijden N, Delputte PL, Favoreel HW, Vandekerckhove J, Van Damme J, et al (2003) Involvement of sialoadhesin in entry of porcine reproductive and respiratory syndrome virus into porcine alveolar macrophages. J Virol 77: 8207–8215. 12857889 10.1128/JVI.77.15.8207-8215.2003 PMC165228

[69] Cavrois M, Neidleman J, Kreisberg JF, Greene WC (2007) In vitro derived dendritic cells trans-infect CD4 T cells primarily with surface-bound HIV-1 virions. PLoS Pathog 3: e4. 17238285 10.1371/journal.ppat.0030004 PMC1779297

[70] Wiley RD, Gummuluru S (2006) Immature dendritic cell-derived exosomes can mediate HIV-1 trans infection. Proc Natl Acad Sci U S A 103: 738–743. 16407131 10.1073/pnas.0507995103 PMC1334656

[71] Berney C, Herren S, Power CA, Gordon S, Martinez-Pomares L, et al (1999) A member of the dendritic cell family that enters B cell follicles and stimulates primary antibody responses identified by a mannose receptor fusion protein. J Exp Med 190: 851–860. 10499923 10.1084/jem.190.6.851 PMC2195630

[72] Kwan WH, Helt AM, Maranon C, Barbaroux JB, Hosmalin A, et al (2005) Dendritic cell precursors are permissive to dengue virus and human immunodeficiency virus infection. J Virol 79: 7291–7299. 15919883 10.1128/JVI.79.12.7291-7299.2005 PMC1143643

[73] Zou Z, Chastain A, Moir S, Ford J, Trandem K, et al (2011) Siglecs facilitate HIV-1 infection of macrophages through adhesion with viral sialic acids. PLoS One 6: e24559. 21931755 10.1371/journal.pone.0024559 PMC3169630

[74] Zhu P, Liu J, Bess J Jr, Chertova E, Lifson JD, et al (2006) Distribution and three-dimensional structure of AIDS virus envelope spikes. Nature 441: 847–852. 16728975 10.1038/nature04817

[75] O'Reilly MK, Paulson JC (2010) Multivalent ligands for siglecs. Methods Enzymol 478: 343–363. 20816489 10.1016/S0076-6879(10)78017-4 PMC3012384

[76] Stacey AR, Norris PJ, Qin L, Haygreen EA, Taylor E, et al (2009) Induction of a striking systemic cytokine cascade prior to peak viremia in acute human immunodeficiency virus type 1 infection, in contrast to more modest and delayed responses in acute hepatitis B and C virus infections. J Virol 83: 3719–3733. 19176632 10.1128/JVI.01844-08 PMC2663284

[77] Pitha PM (2011) Innate antiviral response: role in HIV-1 infection. Viruses 3: 1179–1203. 21994776 10.3390/v3071179 PMC3185785

[78] Sagar M, Akiyama H, Etemad B, Ramirez N, Freitas I, et al (2012) Transmembrane Domain Membrane Proximal External Region but Not Surface Unit-Directed Broadly Neutralizing HIV-1 Antibodies Can Restrict Dendritic Cell-Mediated HIV-1 Trans-infection. J Infect Dis 205: 1248–1257. 22396600 10.1093/infdis/jis183 PMC3308909

[79] Sherer NM, Lehmann MJ, Jimenez-Soto LF, Ingmundson A, Horner SM, et al (2003) Visualization of retroviral replication in living cells reveals budding into multivesicular bodies. Traffic 4: 785–801. 14617360 10.1034/j.1600-0854.2003.00135.x

[80] Dohi T, Nores G, Hakomori S (1988) An IgG3 monoclonal antibody established after immunization with GM3 lactone: immunochemical specificity and inhibition of melanoma cell growth in vitro and in vivo. Cancer Res 48: 5680–5685. 3167827

