# Supplementary figures and images for: Interferon-Inducible Mechanism of Dendritic Cell-Mediated HIV-1 Dissemination Is Dependent on Siglec-1/CD169
Source: PLoS Pathog. 2013 Apr 11;9(4):e1003291. doi: 10.1371/journal.ppat.1003291 (PMC3623718; doi:10.1371/journal.ppat.1003291)

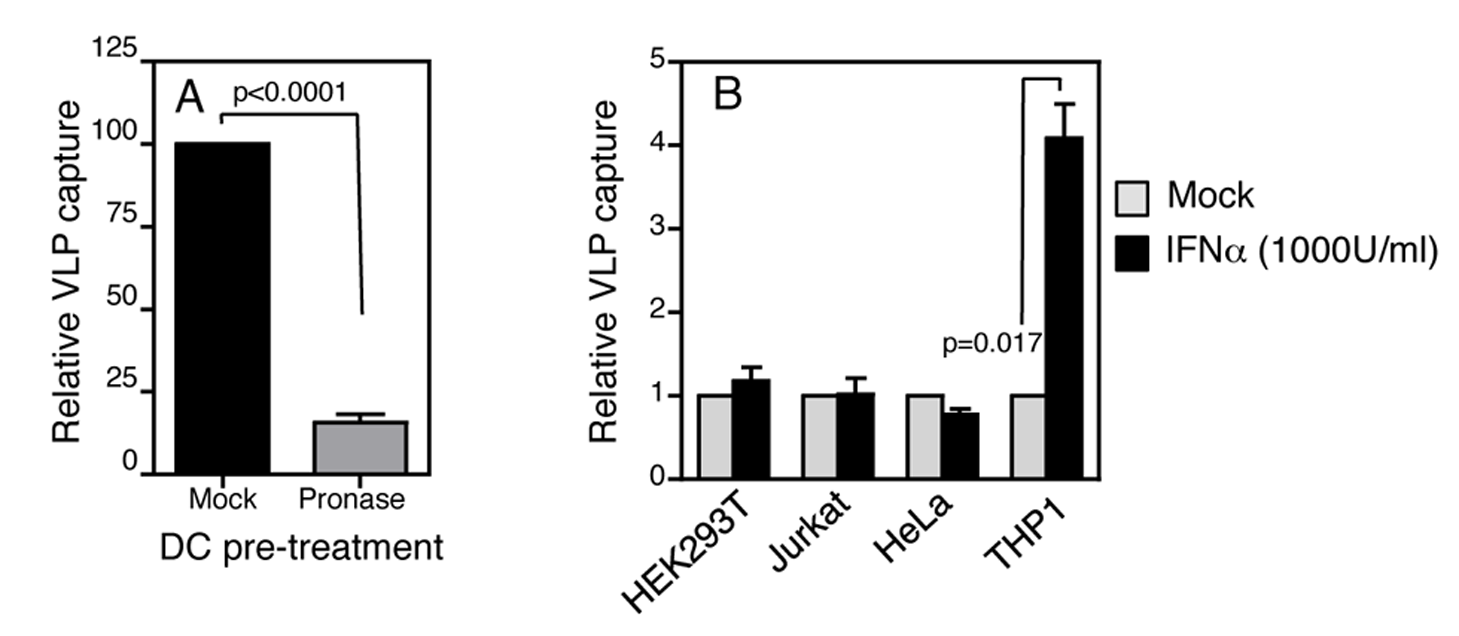

Supplement: Figure S1 — GSL-dependent HIV-1 capture mechanism is protease sensitive and is specific to myeloid cells. A. Gag-eGFP VLP capture assays were performed on mature DCs untreated (mock) or treated with 2 mg/ml pronase prior to HIV Gag-eGFP VLP exposure. B. HIV Gag-eGFP VLP capture by cells untreated (mock) or treated with IFNα (1000 U/ml) for 48 h prior to VLP exposure. Reported data in panels A and B are relative capture normalized to mock treated cells (from 4 independent experiments; mean +/−SD). (TIF) [file ppat.1003291.s001.tif]

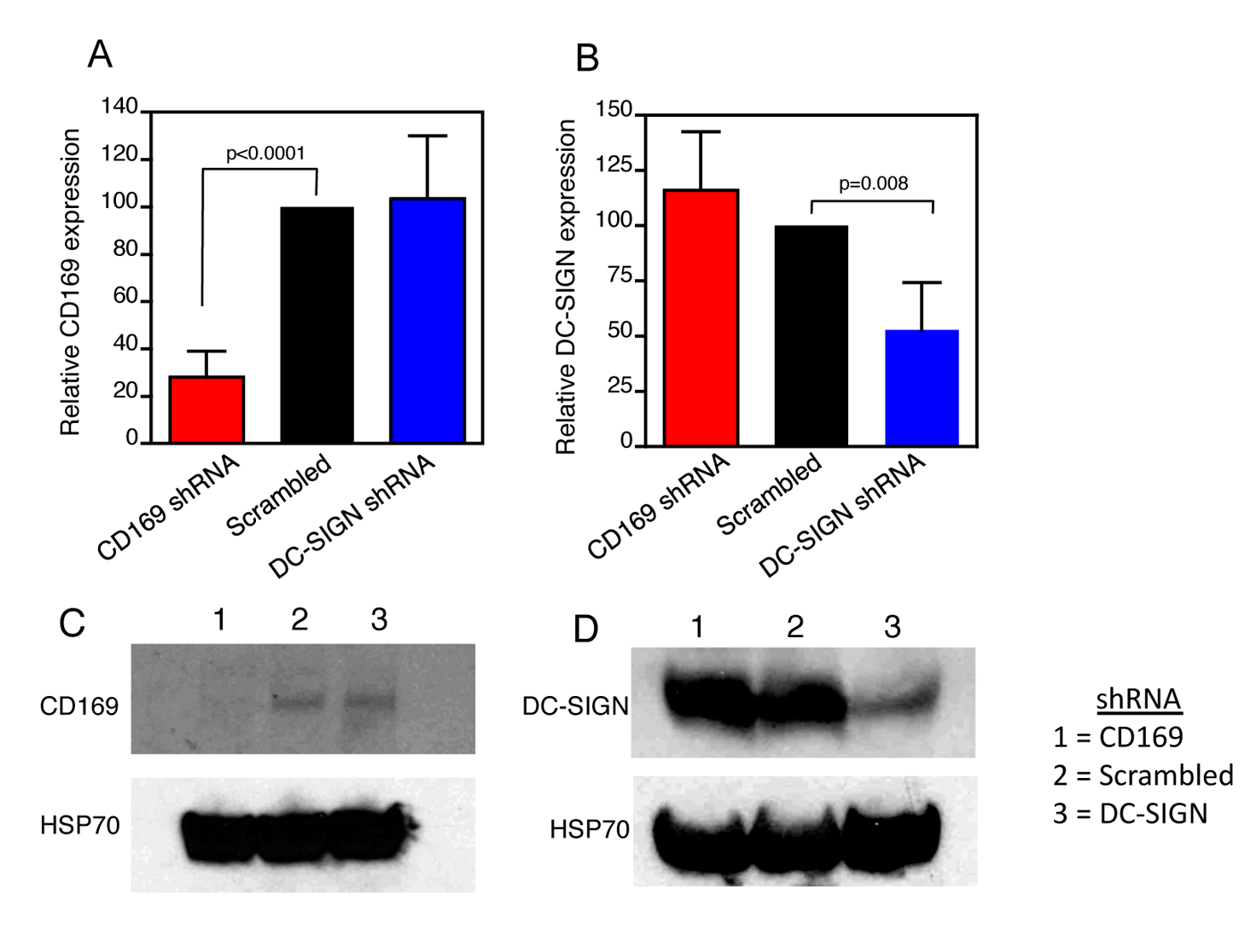

Supplement: Figure S2 — Selective depletion of HIV-1 attachment factor expression in dendritic cells. Immature DCs transduced with shRNAs expressing lentivectors targeting DC-SIGN, CD169, or scrambled sequence were stimulated with LPS (100 ng/ml) for 48 h and assayed for cell surface expression by FACS (A, B) or total cellular expression by western blot analysis (C, D) of CD169 (A, C) or DC-SIGN (B, D). Cell surface expression of CD169 (A) or DC-SIGN (B) is reported as relative MFI expression to that of cells transduced with lentivectors expressing scrambled shRNA, and is the average of three independent experiments (mean ± SD). (TIF) [file ppat.1003291.s002.tif]

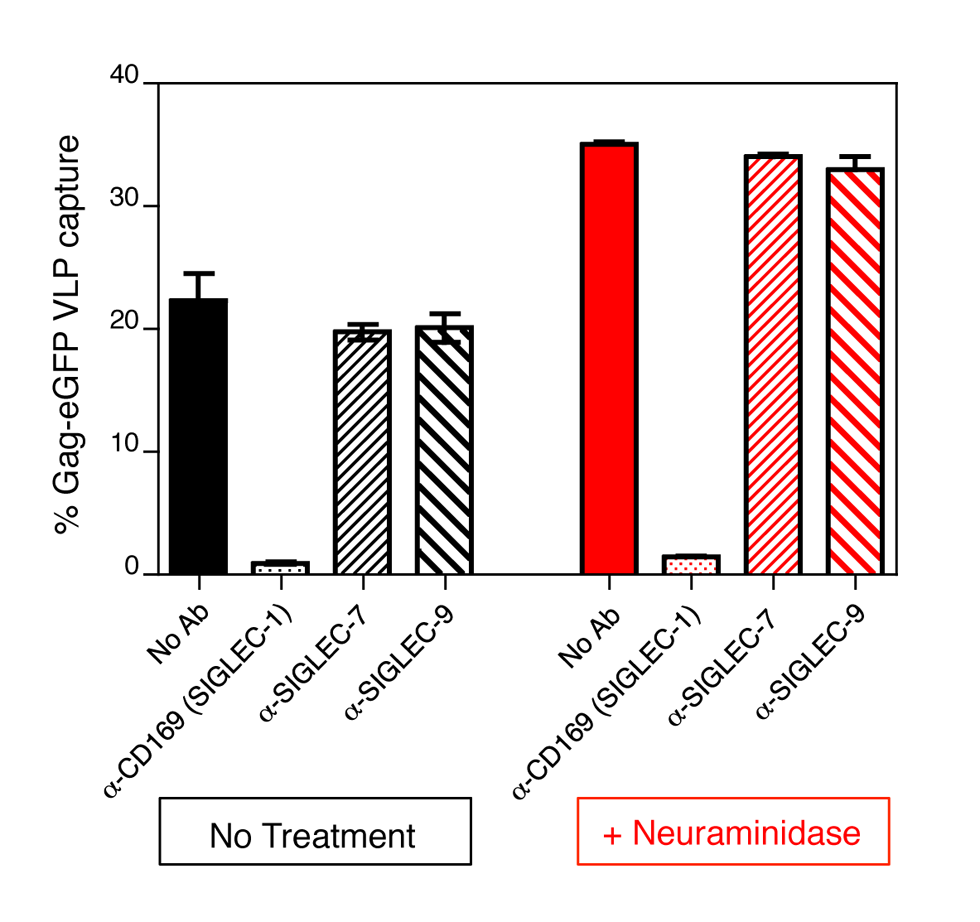

Supplement: Figure S3 — CD169 is the sole SIGLEC family member responsible for HIV-1 capture by dendritic cells. Mature DCs, left untreated or pre-treated with neuraminidase, were incubated with 1 µg of antibody directed against CD169 (Siglec-1), Siglec-7, or Siglec-9. Capture assays with HIV Gag-eGFP VLPs were performed in duplicate on mature DCs from two independent donors, and the average Gag-eGFP VLP capture +/− SD is reported. (TIF) [file ppat.1003291.s003.tif]

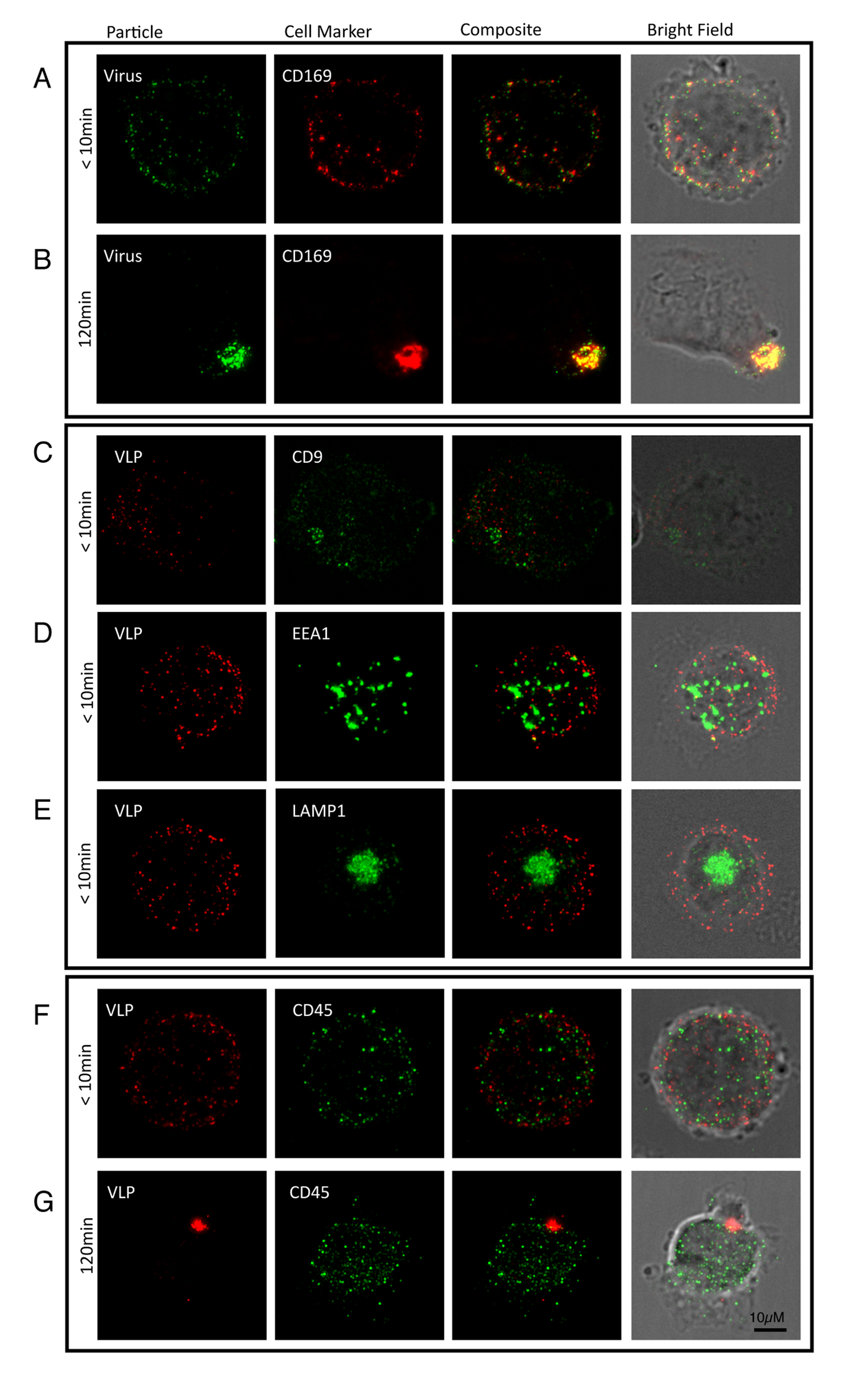

Supplement: Figure S4 — HIV-1 particles captured by mature DCs are co-localized with CD169. (A) Co-localization of HIV/Lai-iGFP (green) with CD169 (red) on mature DC surface within 10 minutes of virus exposure, (B) and in peripheral polarized compartment upon 120 minutes of virus exposure. (C–G) Mature DCs incubated with Gag-mCherry VLP (red) for <10 minutes were probed for cell surface (CD9) and endosomal markers (EEA1 and LAMP1). Staining of cellular markers was visualized by Alexa488-conjugated secondary antibodies (green); representative images are shown for staining with (C) CD9, (D) EEA1 and (E) LAMP1. Lack of co-localization between CD45 (green) and HIV Gag-mCherry VLP in mature DCs after 10 min (F) or 120 min (G) post virus exposure. (TIF) [file ppat.1003291.s004.tif]

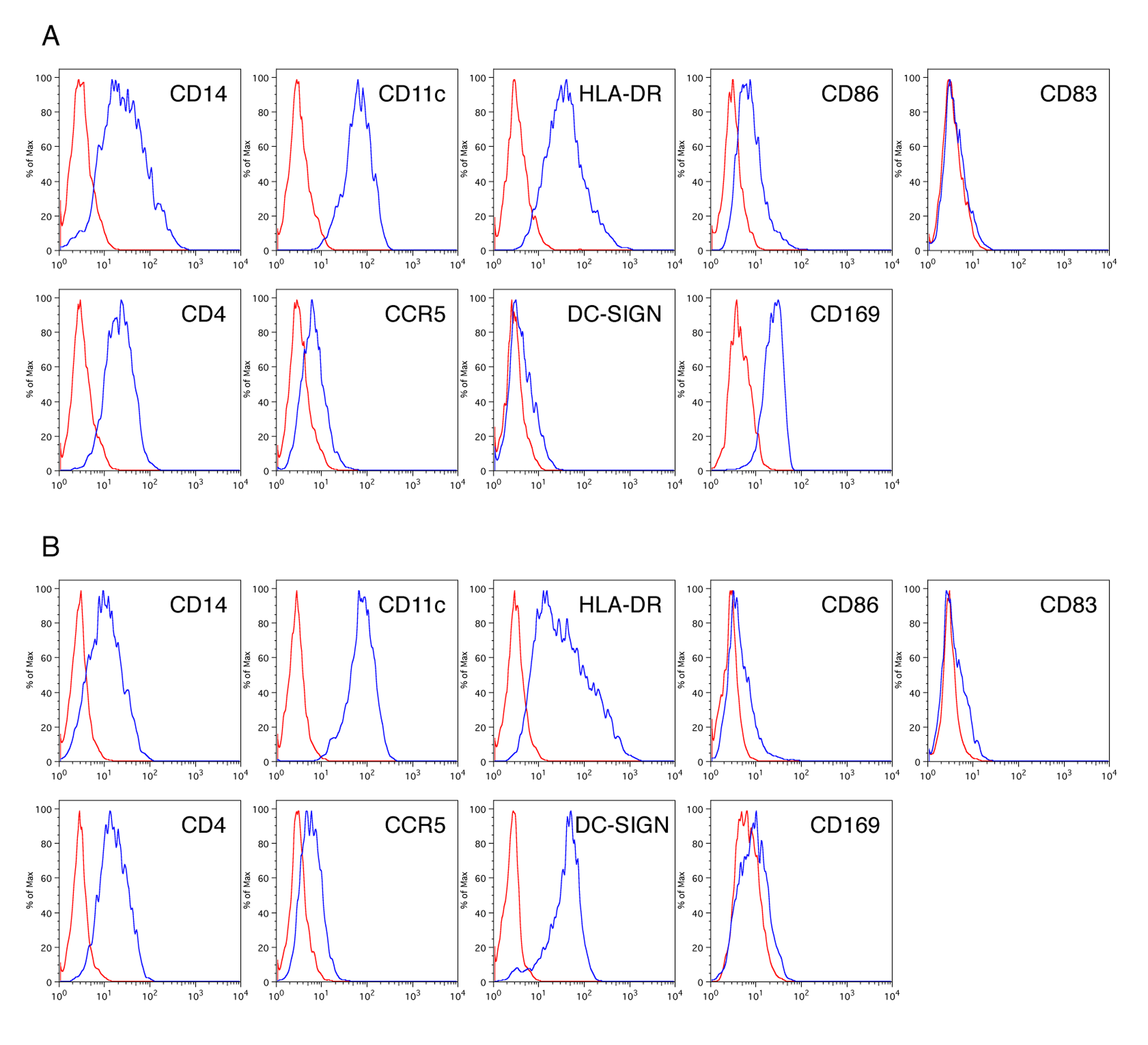

Supplement: Figure S5 — Differential expression of CD169 and DC-SIGN on IFN-α and IL4 differentiated DCs. Immunophenotypic characterization of IFN-DCs (GM-CSF + IFNα 3 days post initiation of differentiation) (A) and IL4-DCs (GM-CSF + IL-4, 3 days post-initiation of differentiation) (B) was determined by FACS analysis. The red histograms represent staining with the isotype control antibody and the blue histograms represent staining for antibodies to the specific cell surface markers. (TIF) [file ppat.1003291.s005.tif]

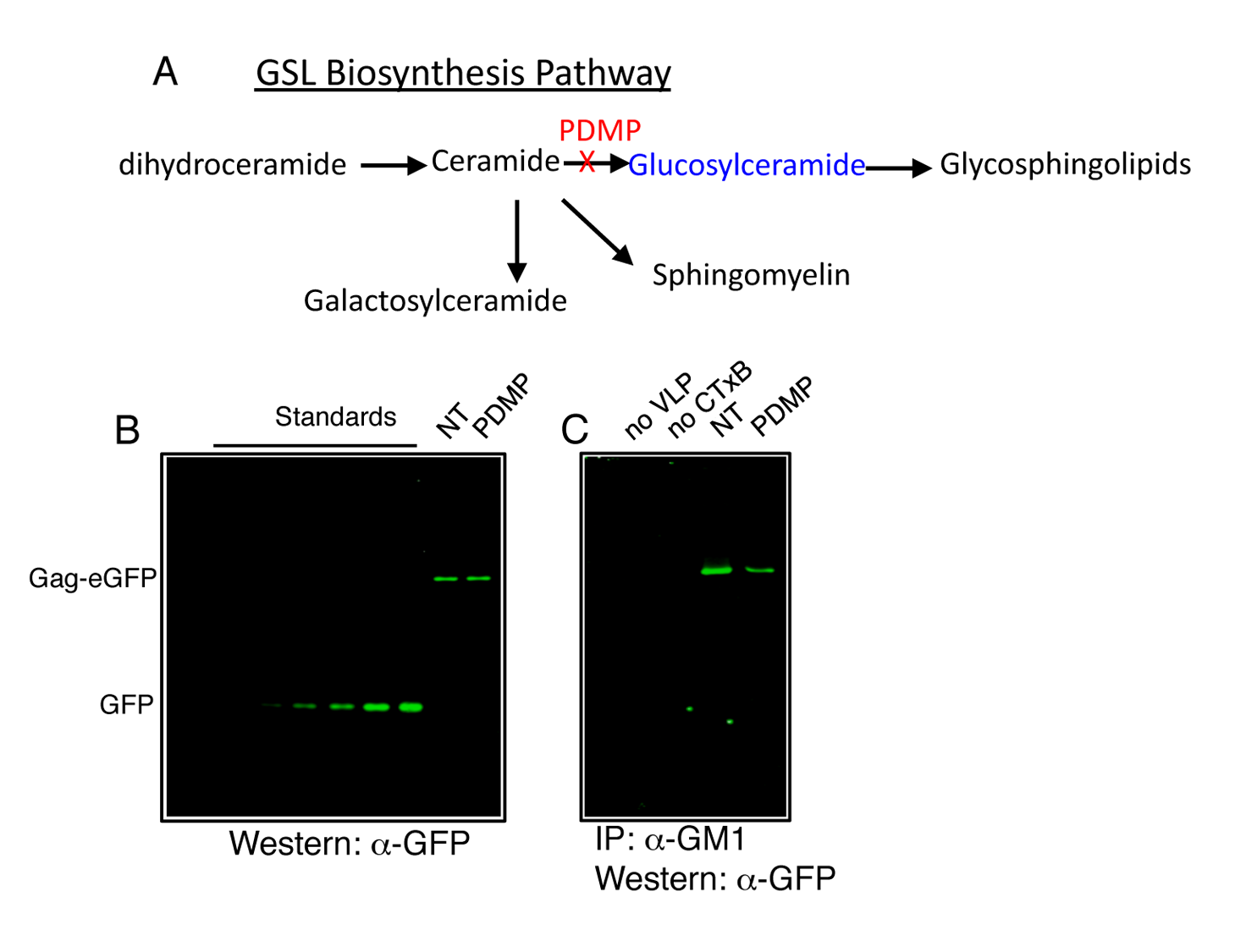

Supplement: Figure S6 — HIV Gag-eGFP VLPs produced from PDMP-treated HEK293T cells are depleted in GSLs. The model depicts the simplified GSL biosynthesis pathway, and the enzymatic step (synthesis of glucosylceramide, catalyzed by the enzyme, glucosylceramide synthase) inhibited by the cationic lipid, PDMP (A). The amount of HIV Gag-eGFP VLPs produced from transient transfection of HEK293T cells in the presence or absence (NT) of PDMP (10 µM), is quantified by quantitative LICOR-western blot analysis (B) using a α-GFP polyclonal antibody. The relative incorporation of GSLs in VLPs derived from untreated (NT) or PDMP-treated HEK293T cells were determined by immunoprecipitation with biotin-conjugated CtxB and streptavidin-dynabeads. Quantification of the immunoprecipitated virus particles was enabled by quantitative western blot analysis using a α-GFP polyclonal antibody (C). (TIF) [file ppat.1003291.s006.tif]

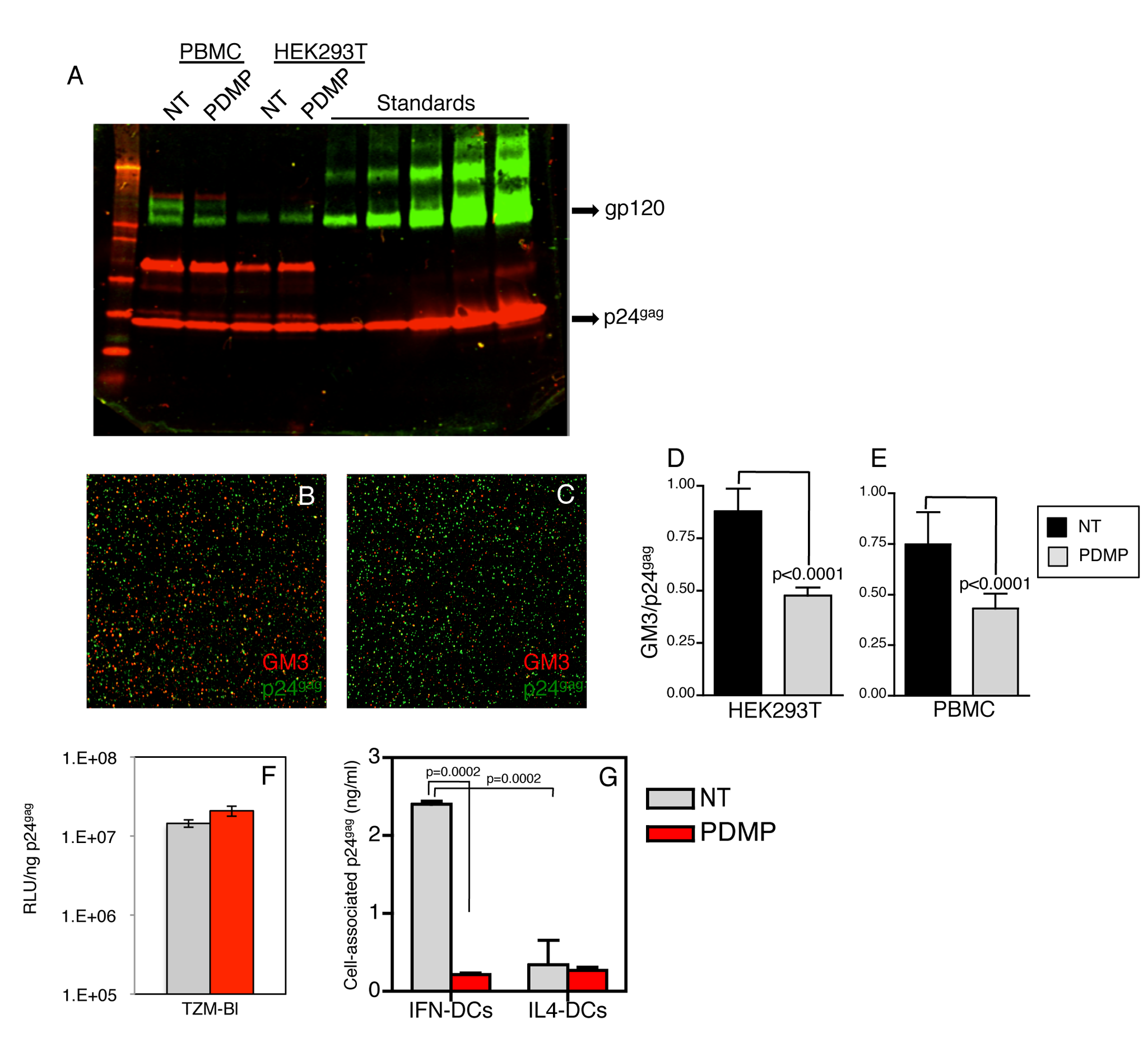

Supplement: Figure S7 — Depletion of GSLs from HEK293T or PBMC-derived HIV-1 particles attenuates virus capture by IFN-DCs. A. HIV-1 Env (gp120) and p24gag content of HIV/Lai-Bal virus particles derived from HEK293T or PBMCs in the absence (NT) or presence of PDMP (10 µM), was determined by quantitative LICOR-western blot analysis using α-gp120 and α-p24gag primary antibodies and IR680 and IR800-conjugated secondary antibodies, respectively. Virions (HIV/Lai-Bal) derived from untreated (B) or PDMP-treated (C) PBMCs were labeled for p24gag (green) and GM3 (red). Representative fields are shown and the average mean fluorescence intensity of GM3 normalized to p24gag ± SD is reported for HEK293T (D) and PBMC-derived (E) virus stocks. F. Infectivity of HIV/Lai-Bal derived from PBMCs in the absence (NT) or presence of PDMP (10 µM) was determined on TZM-bl reporter cells. G. Capture assays with IFN-DCs and IL4-DCs were performed with PBMC-derived HIV/Lai-Bal (±PDMP) and cell-associated p24gag content determined by ELISA. Data reported is average of three independent experiments, +/− SD. (TIF) [file ppat.1003291.s007.tif]

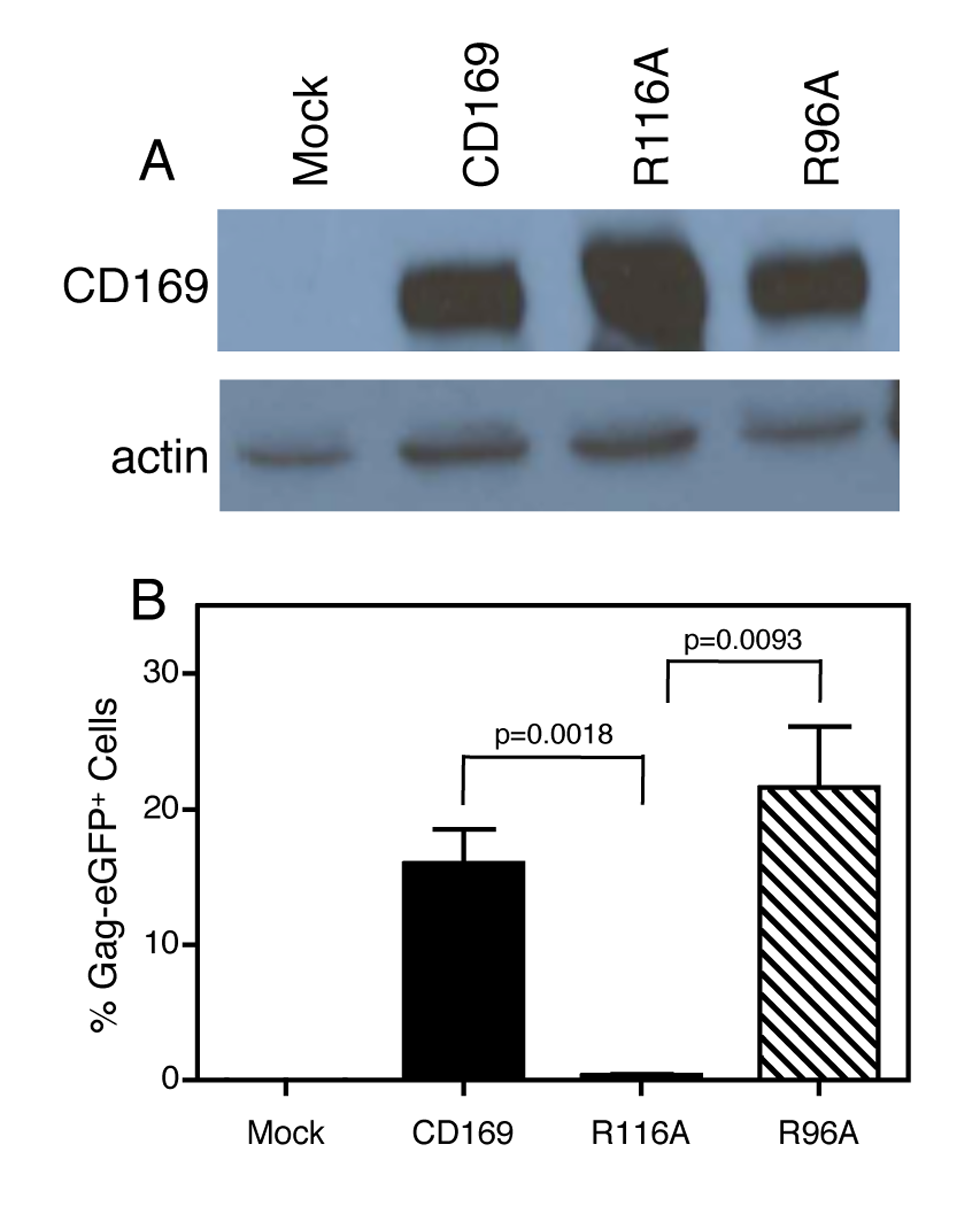

Supplement: Figure S8 — Mutation of the sialic acid recognition motif in CD169 abrogates HIV-1 capture. Expression of CD169 or mutants, R96A and R116A, in transiently transfected HEK293T cells was determined by western blot analysis (A). The percentage of CD169 (or mutant) positive cells capturing HIV Gag-eGFP VLPs was determined by FACS analysis (B). The data reported is the average of two independent experiments performed in triplicate (mean ± SD). (TIF) [file ppat.1003291.s008.tif]

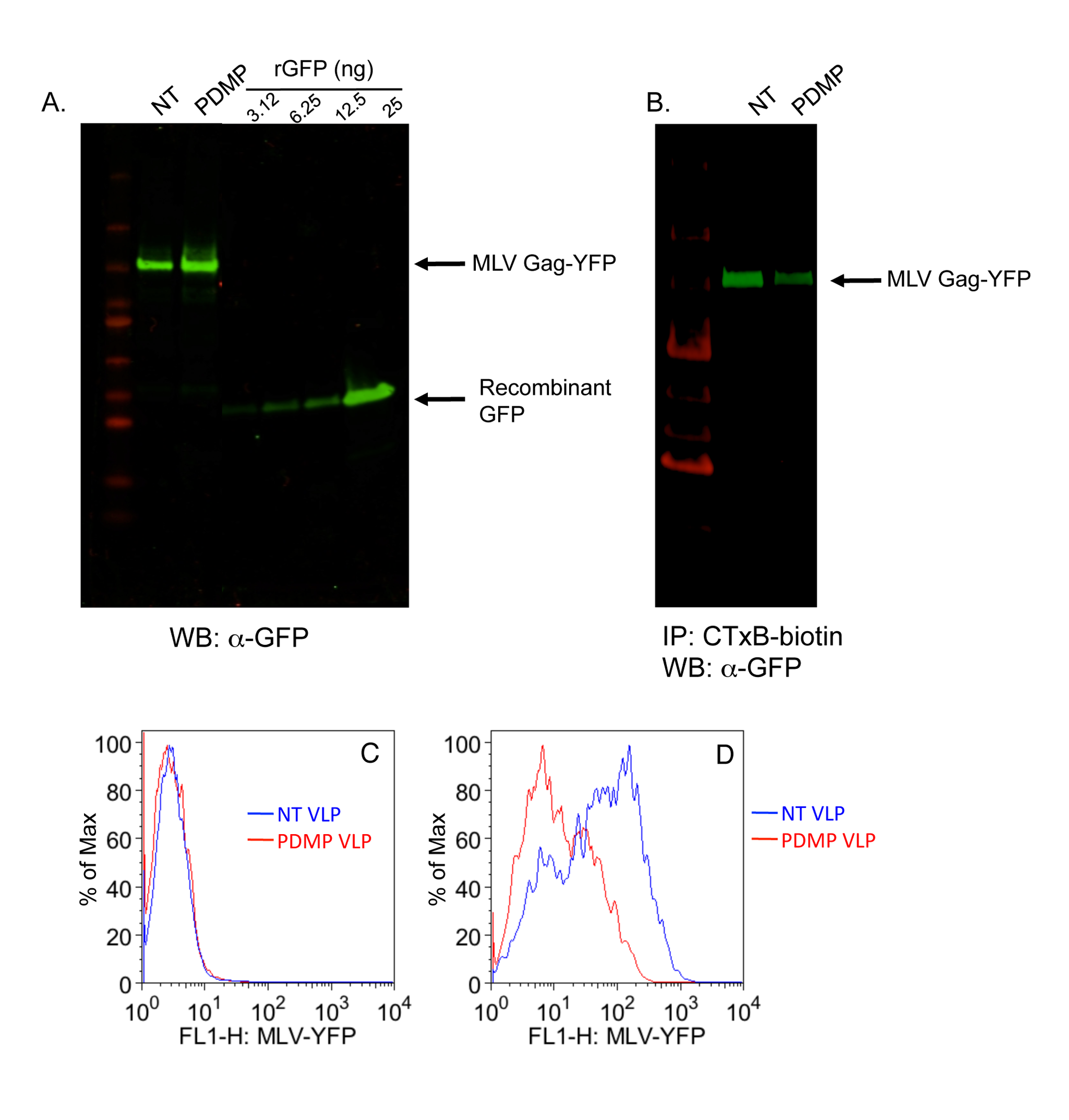

Supplement: Figure S9 — Characterization of MLV Gag-YFP VLPs. (A) The amount of MLV Gag-YFP VLPs produced from transient transfection of untreated (NT) or PDMP (10 µM) treated HEK293T cells was quantified by quantitative LICOR-western blot analysis using an α-GFP polyclonal antibody and IRDye 800CW-conjugated donkey α-goat-IgG secondary antibody (B) and α-MLV Gag monoclonal antibody. B. The relative incorporation of GSLs in MLV Gag-YFP VLPs derived from untreated (NT) or PDMP-treated HEK293T cells were determined by immunoprecipitation with biotin-conjugated CtxB and streptavidin-dynabeads. Quantification of the immunoprecipitated virus particles was enabled by quantitative western blot analysis using an anti-GFP polyclonal antibody. C. Capture of MLV Gag-YFP VLPs (±PDMP) by Raji (C) and Raji/CD169 cells (D) was determined by measuring the percentage of YFP positive cells by FACS. The data reported is representative of two independent experiments performed in duplicate. (TIF) [file ppat.1003291.s009.tif]
